# *Meso*-functionalization of calix[4]arene with 1,3,7-triazapyrene in the design of novel fluorophores with the dual target detection of Al^3+^ and Fe^3+^ cations†

**DOI:** 10.1039/d0ra10605d

**Published:** 2021-02-04

**Authors:** Timofey D. Moseev, Igor A. Lavrinchenko, Mikhail V. Varaksin, Diana Yu. Pobedinskaya, Oleg P. Demidov, Ivan V. Borovlev, Valery N. Charushin, Oleg N. Chupakhin

**Affiliations:** Ural Federal University 19 Mira Str. 620002 Ekaterinburg Russia m.v.varaksin@urfu.ru; Institute of Organic Synthesis, Ural Branch of the Russian Academy of Sciences 22 S. Kovalevskaya Str. 620990 Ekaterinburg Russia chupakhin@ios.uran.ru; North Caucasus Federal University 1 Pushkin Str. 355009 Stavropol Russia

## Abstract

A *meso*-functionalization strategy has successfully been applied to the synthesis of novel 1,3,7-triazapyrene derivatives of calixarenes. The key synthetic step in these transformations providing the direct C–C bond formation is nucleophilic substitution of hydrogen (S_N_^H^) in 1,3,7-triazapyrene. General photophysical characteristics for these macrocyclic compounds, as well as features in emission properties upon addition of various metal cations have been elaborated. Studies using NMR spectroscopy have also shown a mutual effect of both calix[4]arene and 1,3,7-triazapyrene moieties on the coordination process. The complex stoichiometry and binding constants for Al^3+^ and Fe^3+^ guests have been explored with titration experiments.

## Abstract 2

Novel bifunctional fluorophores with the dual target detection of Al^3+^ and Fe^3+^ cations were synthesized in yields up to 70% *via* bridge-functionalization of calix[4]arenes with the 1,3,7-triazapyrene scaffold.

## Graphical abstract

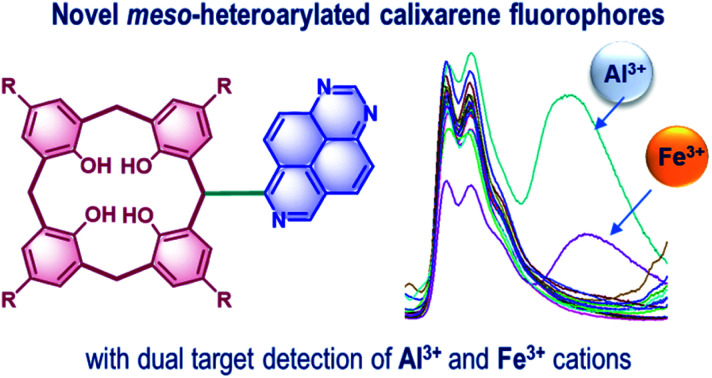

## Introduction

Over the last decade, the design of organic molecules for selective detection of biologically and environmentally important analytes, such as small molecules and metal ions attracts a considerable attention from the broad scientific community.^1^ Among a variety of metal ions, Al^3+^ and Fe^3+^ are known to be of great importance due to a wide range of practical applications in machinery, aviation, electronic devices, and daily life.^2^ Aluminum-containing substances are well-spread in food additives and water treatment systems.^3^ It is worth noting that accumulation of aluminum in living system turns out to cause anemia and pathology of the nervous system, *e.g.*, Alzheimer disease.^4^ Besides, iron is an important microelement that plays a key role in the metabolism, synthesis of DNA and RNA,^5^ also it provides the ability of haem to transfer oxygen.^6^ On the one hand, the lack of Fe^3+^ ions results in hemochromatosis, liver injury, Parkinson's disease, and cancer. On the other hand, its abundance is accompanied by certain types of cancer, dysfunction of the heart or pancreas.^7^ In this regard, the elaboration of efficient chemosensors affording prompt monitoring and the quantitative assessment of ions in biological and environmental systems seems to be a challenging task for modern organic chemistry and materials science.

Calixarenes and their functional derivatives are known to be of practical interest as efficient macrocyclic ionophore receptors. Due to their unique three-dimensional characteristics and also diverse opportunities for the target functionalization, these molecules are widely used as catalysts, liquid crystals, and fluorescent materials.^8^ Also, macrocyclic ring of calixarene is considered to be a promising scaffold in the design of active pharmaceutical ingredients, drug delivery systems, and modern chemosensors.^9^ Moreover, calixarene-based compounds are known to be used as highly efficient sensory systems for selective detection of various metal ions (Al^3+^, Fe^3+^, Sn^2+^, Hg^2+^, Cd^2+^, *etc.*).^10^

Notably, there have been various approaches for the calixarene scaffold functionalization both on upper and low rims, whereas modification of the methylene bridges has remained relatively unexplored for a long time.^11^ Incorporation of functional blocks into this position enables one to alter solubility and flexibility, control the conformational preferences of the macrocyclic ring, and also provides the point for connection to surfaces or other molecules. Bridge-functionalized calixarenes can be obtained through either cyclocondensation of substituted phenols or *via* direct modification of the methylene groups.^12^ One of these approaches is lithiation of the methylene carbon followed by direct coupling with electrophiles^13^ to give the corresponding *meso*-substituted calixarenes. In addition, synthetic opportunities for the C–Li/C–H coupling reactions of lithiated calix[4]arene with π-deficient azaaromatics are able to be considered as fruitful and challenging ones. These green chemistry-oriented transformations based on nucleophilic substitution of hydrogen (S_N_^H^) in azines have recently been shown to be efficient methods for the synthesis of mono-, di- and triazinyl-substituted tetramethoxycalixarenes.^14^

This paper deals with a convenient synthetic methodology to afford novel calix[4]arenes modified at the bridge position with azaaromatic scaffold, namely 1,3,7-triazapyrene. An increased interest in this type of macroheterocyclic structures is due to wide opportunities for their practical applications in molecular electronics, *e.g.*, organic photovoltaics (OPVs), organic light-emitting diodes (OLEDs), and organic field-effect transistors (OFETs) as well.^15^ Besides, some intriguing results on photophysical properties of novel 1,3,7-triazapyrene-modified calixarenes are reported herein, as well as their possibilities for practical use as effective macrocyclic sensory molecules for metal ions, in particular for the dual detection of Al^3+^ and Fe^3+^ cations.

## Results and discussion

**Scheme 1:**
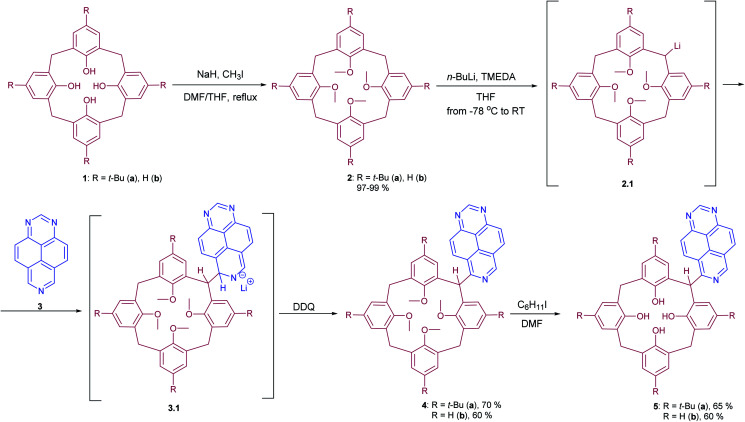
*Meso*-heteroarylation strategy in the synthesis of bridge-modified calix[4]arenes.

In order to synthesize the desired 1,3,7-triazapyren-6-yl derivatives of calix[4]arenes, a *meso*-functionalization strategy including a series of subsequent transformations, such as protection of the hydroxy group, bridge-lithiation, followed by nucleophilic substitution of hydrogen (S_N_^H^) in 1,3,7-triazapyrene, oxidation of the σ^H^-adducts, and final deprotection has successfully been applied in this study (Scheme 1). It should be emphasized that the exploited reactions of nucleophilic substitution of hydrogen (S_N_^H^) are now considered as environmentally benign PASE (Pot, Atom, Step Economy) methodology,^16^ enabling the direct C–H functionalization of aromatics,^17^ as well as non-aromatic azaheterocyclic substrates.^18^ At the same time, a limited number of methods for the synthesis of substituted 1,3,7-triazapyrene derivatives are now available.^19^ Taking into account this issue, the S_N_^H^ methodology has herein been chosen as a basic synthetic approach towards to *meso*-functionalized calix[4]arenes.

Initially, to protect the hydroxy group in calix[4]arene, we exploited the known approach.^20^ The further direct C–Li/C–H coupling reaction of *meso*-Li-calix[4]arene with 1,3,7-triazapyrene was carried out according to the modified literature procedure.^14^ The methylene group of calix[4]arene 2 was first lithiated with *n*-BuLi and TMEDA, the lithium compound 2.1 reacted subsequently with N=C–H fragment of 1,3,7-triazapyrene 3, thus leading to unstable σ-adduct 3.1. These intermediates could be transformed into the corresponding products 4 in 60–70% yields by action of DDQ as oxidizing agent. Notably, the typical procedure^21^ for demethylation using BBr_3_ in DCM was experimentally found to result in either the partial deprotection (formed in the complex mixtures of various isomers) or decomposition of starting materials as a result of breaking C–C bond between bridge calixarene and azaheterocyclic moieties. The latter was the reason to apply another demethylation procedure using by iodocyclohexane in DMF.^22^ In this case, tetrahydroxycalix[4]arenes 5 modified with the 1,3,7-triazapyrene scaffold at the *meso*-position have finally been synthesized in 60–65% yields.

Structures of the bridge-heteroaryl-substituted tetramethoxy and tetrahydroxy derivatives of calix[4]arenes were confirmed by ^1^H, ^13^C NMR, and IR spectroscopy including two-dimension NMR experiments, such as ^1^H–^13^C HSQC, ^1^H–^13^C HMBC, ^1^H–^1^H COSY, as well as by the data of mass spectrometry and elemental analysis.

The synthesized methoxy- (4a, b) or hydroxycalixarenes (5a, b), being species that are devoid of sterically bulky substituents at the oxygen atom, are known to be characterized by a dynamic equilibrium between conformers in solutions. In order to fix a more preferable conformation for these species, the complexation strategy with Na^+^ was used. According to the ^1^H NMR spectra recorded for 4a after addition of NaI, specific changes were found with regard to the signals for aryl and methylene bridge protons. In particular, several peaks are observed in the regions, where the methylene (3.0–4.5 ppm) and aryl (6.4–7.8 ppm) proton signals are registered in the absence of sodium ions. After addition of Na^+^ ions, the methylene protons resonated as two signals (4H) at 3.4 ppm, one signal (2H) overlapped with ones of methyl groups at 4.27 ppm, and one signal (1H) at 7.72 ppm. It is worth noting that these features observed correlate well with the literature data for the structurally similar *meso*-functionalized calix[4]arenes reported.^23^ Besides, there is one doublet (6H) and one singlet (2H) found in the aryl region of the NMR spectra. Thus, we suppose that partial cone (paco) is the most favourable conformation for the synthesized calixarenes in the presence of Na^+^ ions. This suggestion is also supported by the data for the bridge-substituted calixarenes with bulky fragments at the *meso*-position.^24^

**Fig. 1:**
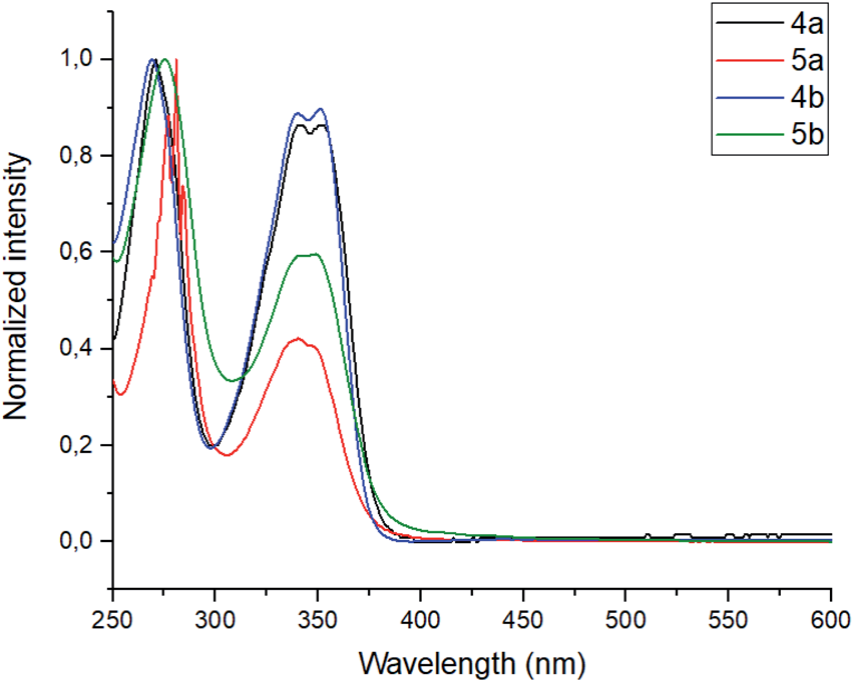
Normalized absorbance spectra of calixarenes 4 and 5. Sample preparation: *C* = 1.0 × 10^−5^ mol L^−1^ in THF at room temperature.

The photophysical properties, such as absorbance and emission spectra of azapyrene-modified calix[4]arenes 4 and 5 have comprehensively been studied to evaluate opportunities for their practical applications in the design of prospective fluorescent sensors (Table 1). As far as the absorbance spectra are concerned, the obtained calix[4]arenes 4 and 5 have proven to possess a similar pattern, exhibiting two broadened bands with maxima at 275 and 350 nm in a THF solution (Fig. 1).

**Table 1:** Photophysical properties of *meso*-heteroarylated calix[4]arenes 4 and 5

Entry	Compounds	Absorbance *λ*_abs_^a^ (nm)	Emission *λ*_em_^b^ (nm)
1	4a	270, 340, 350	392, 575 (br)
2	4b	270, 340, 350	391, 467, 498, 550 (br)
3	5a	277, 281, 284, 340 (br)	378, 452, 475
4	5b	275, 345 (br)	390, 451, 476

a Absorbance maxima in THF.

b Emission maxima in THF.

**Fig. 2:**
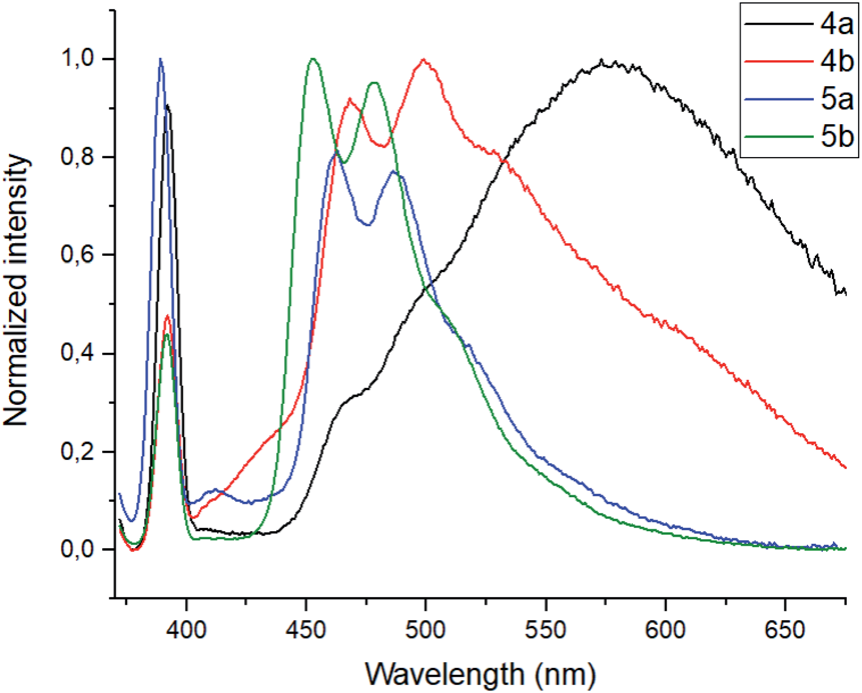
Normalized emission spectra for calixarenes 4 and 5. Conditions: 4a: *λ*_ex_ = 352 nm, 4b: *λ*_ex_ = 351 nm, 5a: *λ*_ex_ = 341 nm, 5b: *λ*_ex_ = 350 nm. Sample preparation: *C* = 1.0 × 10^−5^ mol L^−1^ in THF at room temperature.

In the emission spectra of tetramethoxy-substituted calix[4]arenes 4, the intramolecular charge transfer (ICT) effects are observed as bands with maxima at 575 nm and 550 nm for 4a and 4b, respectively (Fig. 2).^25^ Notably, the calix[4]arene scaffold is likely to act here as an electron-donating group, whereas 1,3,7-triazapyrene plays a role of electron-withdrawing functional block. Moreover, compound 4a is characterized by the enhanced ICT effect due to a strong inductive electronic effect (+I) of *tert*-butyl substituent in the *para*-positions of the benzene rings. In case of tetrahydroxy substituted calixarenes 5, a complicated structure of peaks is presumably attributed to 1,3,7-triazapyrene contribution in the range from 450 to 550 nm, observed in the emission spectra.

**Fig. 3:**
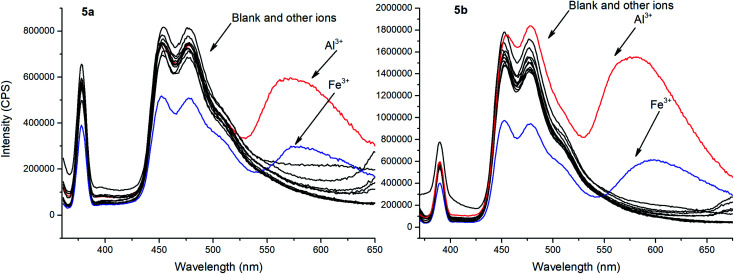
Emission spectra of 5a and 5b with the presence of various metal ions (10 equiv.). Sample preparation: *C* = 1.0 × 10^−5^ mol L^−1^ in THF at room temperature.

To evaluate ionophore ability and selectivity of the obtained fluorophores, the emission spectra taken upon the addition of various metal cations, such as Al^3+^, Ba^2+^, Be^2+^, Ca^2+^, Co^2+^, Cu^2+^, Fe^3+^, K^+^, Mg^2+^, Ni^2+^, Sr^2+^, Zn^2+^, Na^+^, Bi^3+^, Cd^2+^, Hg^2+^, Pb^2+^, Sn^2+^ have also been investigated. It is worth mentioning that tetramethoxy-substituted calix[4]arenes 4a, b showed no selectivity to metal cations (see ESI S39 and S40†). At the same time, additional peaks in the emission spectra at 560 nm and 570 nm, respectively, have been observed in case of tetrahydroxycalix[4]arene 5a and 5b in the presence of Al^3+^ and Fe^3+^ cations (Fig. 3). In this regard, the chelation of iron(iii) ions might induce both appearance of additional peaks, but also decrease in the emission intensity. The latter phenomenon could be accounted for the oxidation of phenolic fragments of calix[4]arenes into the corresponding quinones by action of Fe^3+^ ions.^26^

**Fig. 4:**
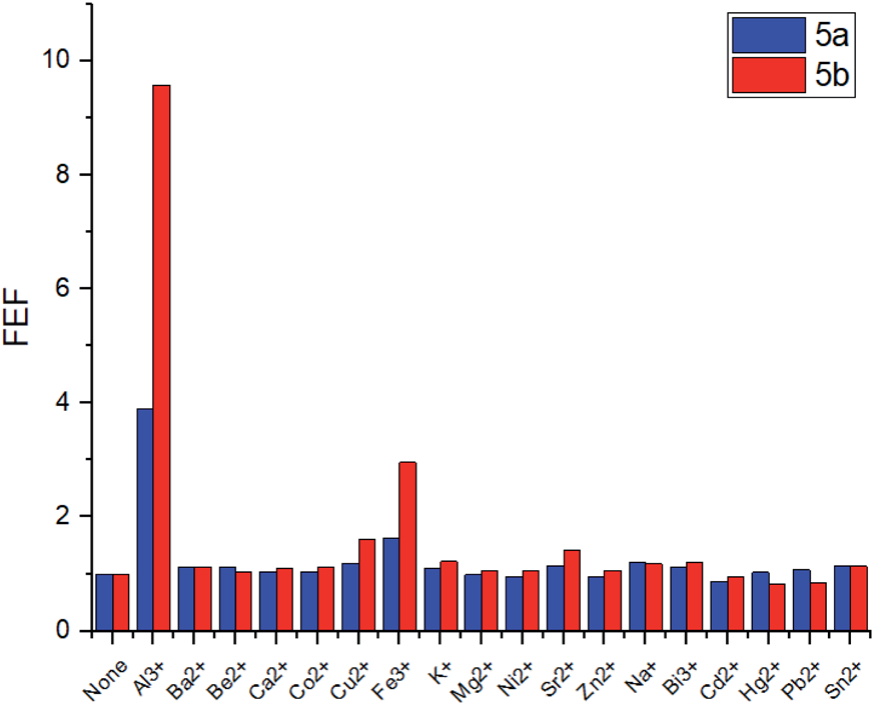
Fluorescence enhancement factors (FEF = *I*/*I*_o_) of 5a and 5b upon addition of various metal cations. *I*_o_ = fluorescence emission intensity of free 5a or 5b, *I* = fluorescence emission intensity of 5a or 5b with metal ion respectively. Conditions: 5a: *C*_5a_ = 10^−5^ mol L^−1^ in THF, 10 equiv. of metal ion in H_2_O, *λ*_ex_ = 341 nm, *λ*_em_ = 560 nm; 5b: *C*_5b_ = 10^−5^ mol L^−1^ in THF, 10 equiv. of metal ion in H_2_O, *λ*_ex_ = 350 nm, *λ*_em_ = 570 nm.

To evaluate the changes in emission intensities for the obtained compounds in the presence of various metals cations quantitatively, the fluorescence enhancement factors (FEF = *I*/*I*_o_) have been calculated according to the typical procedure (Fig. 4).^27^ In case of compound 5a, the emission intensity (*λ*_em_ = 560 nm) appears to be four times higher in the presence of Al^3+^ and 1.5 times higher with Fe^3+^ than in the blank experiment performed. Moreover, the emission intensity (*λ*_em_ = 570 nm) for 5b with Al^3+^ has been found to be 9.5 times higher and 3 times higher in the presence of Fe^3+^. Thus, one can suggest that the *tert*-butyl radical in the *para*-position of the phenol moiety affects the sensory properties of the triazapyrene-modified calixarenes.

**Fig. 5:**
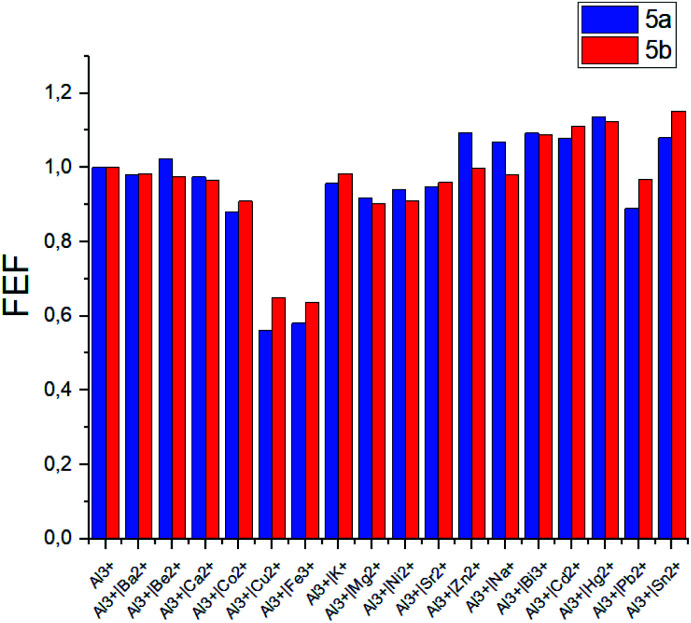
Fluorescence enhancement factors (FEF = *I*/*I*_o_) of 5a and 5b upon addition of various metal cations in competition with Al^3+^. *I*_o_ = fluorescence emission intensity of 5a or 5b with Al^3+^, *I* = fluorescence emission intensity of 5a or 5b with Al^3+^ and metal ion respectively. Experimental conditions: 5a: *C*_5a_ = 10^−5^ mol L^−1^ in THF, 10 equiv. of Al^3+^ and metal ion in H_2_O, *λ*_ex_ = 341 nm, *λ*_em_ = 560 nm; 5b: *C*_5b_ = 10^−5^ mol L^−1^ in THF, 10 equiv. of Al^3+^ and metal ion in H_2_O, *λ*_ex_ = 350 nm, *λ*_em_ = 570 nm.

The selectivity of the *meso*-triazapyrenyl-substituted calix[4]arenes for Al^3+^ ion has also been confirmed by the competition experiment with a number of other ions (Fig. 5). Herein, we have found that emission intensity at the corresponding wavelength in most cases is retained in the presence of other ions in the system. However, emission intensity decreases upon addition of Cu^2+^ ions, probably because of the formation a non-fluorescent copper(ii) complex or dynamic quenching.^28^

**Fig. 6:**
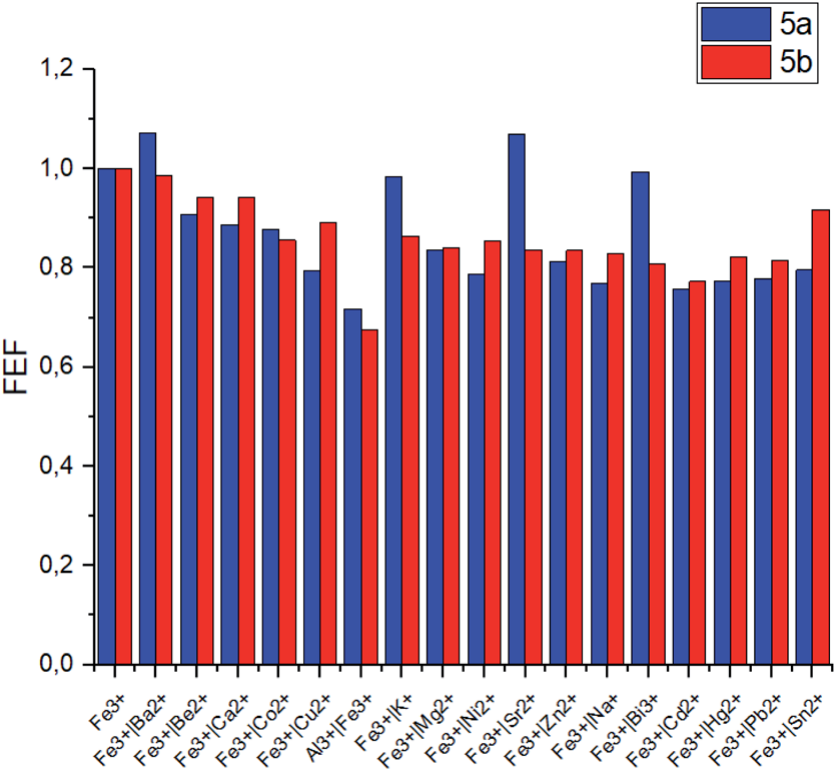
Fluorescence enhancement factors (FEF = *I*/*I*_o_) of 5a and 5b upon addition of various metal cations in competition with Fe^3+^. *I*_o_ = fluorescence emission intensity of 5a or 5b with Fe^3+^, *I* = fluorescence emission intensity of 5a or 5b with Fe^3+^ and metal ion respectively. Experimental conditions: 5a: *C*_5a_ = 10^−5^ mol L^−1^ in THF, 10 equiv. of Fe^3+^ and metal ion in H_2_O, *λ*_ex_ = 341 nm, *λ*_em_ = 560 nm; 5b: *C*_5b_ = 10^−5^ mol L^−1^ in THF, 10 equiv. of Fe^3+^ and metal ion in H_2_O, *λ*_ex_ = 350 nm, *λ*_em_ = 570 nm.

The same competition experiments have been carried out for complexing with Fe^3+^ ions (Fig. 6). Emission intensity at the corresponding wavelength for 5a and 5b has proven to be nearly the same in the presence of other competing ions. It should be noted that the emission intensity decreases slightly, when Cu^2+^ ions are present in the system compared to the previous experiments with Al^3+^. This observation is able to be associated with the enhanced relative stability of calixarene complexes with Fe^3+^ in comparison with the results obtained for non-fluorescent Cu^2+^ complexes.

**Fig. 7:**
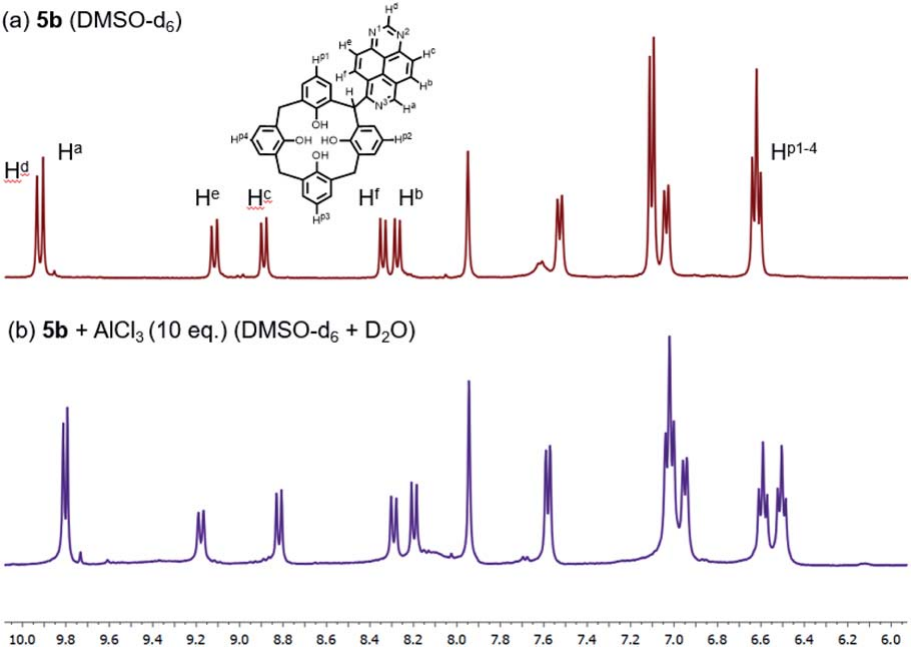
^1^H NMR spectra of 5b in DMSO-*d*_6_ at 298 K (a) free (b) upon addition of AlCl_3_ (10 equiv.) in D_2_O.

To gain insight into the mechanism for interaction of the bridge-1,3,7-triazapyrenyl calix[4]arene ligands with metal cations Al^3+^ and Fe^3+^, a series of NMR experiments have been carried out. We here report that the addition of 10 equivalents of AlCl_3_ in D_2_O to a solution of 5b in DMSO-d_6_ leads to some changes in both chemical shifts and also multiplicities for the signals of phenyl substituents linked directly to C(sp^3^)-functionalized center and 1,3,7-triazapyrene fragments (Fig. 7). Remarkably, it has been found that some 1,3,7 triazapyrene proton signals undergo upfield shifts, while the signals of hydrogen in the *para*-position H^p1-4^ are being split into two multipletes. Based on these changes in the spectra, one can propose that both oxygen atoms of calix[4]arene and nitrogen atoms of 1,3,7-triazapyrene fragment are involved in the organometallic complex formation.

In this study, the Job's method^29^ has been applied to determine the stoichiometry of 5b : Al^3+^ and 5b : Fe^3+^ and shown 1 : 1 host–guest complex formation. Binding interactions of 5b with Al^3+^ and Fe^3+^ cations in THF with addition of various concertation of guests in H_2_O have been estimated using the modified Benesi–Hildebrand equation^30^ (see ESI S27–S30†). We found that K (Al^3+^) = 2.27 × 10^3^ M^−1^; K (Fe^3+^) = 1.63 × 10^5^ M^−1^ corresponding to the literature data for similar fluorescent chemosensors.^8*c*^

## Conclusions

In summary, the *meso*-functionalization strategy, based on the sequence of reactions including protection of the hydroxy group, bridge-lithiation of tetramethoxy-substituted calix[4]arenes followed by C–Li/C–H coupling with 1,3,7-triazapyrene, and further deprotection, has successfully been applied to the synthesis of novel heterocyclic derivatives of calix[4]arenes. The key step in these transformations, which provides a macrocyclic ionophore scaffold to be directly incorporated into the photoactive azaaromatics, is nucleophilic substitution of hydrogen (S_N_^H^) in 1,3,7-triazapyrene. This synthetic approach has been found to result in the formation of new bifunctional fluorophore systems based on *meso*-1,3,7-triazapyrene-substituted calix[4]arene ensembles in good yields under mild reaction conditions. The elucidation of photophysical properties enabled us to reveal the intramolecular charge transfer (ICT) in tetramethoxy *meso*-heteroaryl-substituted calix[4]arenes, which can be considered as challenging molecules for materials science and molecular electronics. The fluorescent sensory properties with respect to metal cations, including applications of *meso*-heteroaryl-substituted calixarenes for the dual target detection of Al^3+^ and Fe^3+^ cations, and also mechanistic features for the complex formation have been studied by NMR spectroscopy. Remarkably, tetrahydroxycalix[4]arenes have experimentally been shown to be more promising compounds in the design of fluorescent chemosensors *versus* the corresponding tetramethoxy analogues. Thus, the properties of azaheterocyclic calix[4]arene fluorophores illustrated in this research indicate new opportunities for practical applications of these macrocyclic ensembles for selective detection of metal ions (Al^3+^, Fe^3+^) in biological, environmental, and technogenic systems.

## Experimental

### General experimental methods

Nuclear Magnetic Resonance (NMR) spectra were recorded on Bruker Avance II (400 MHz) spectrometers. All ^1^H experiments were reported in *δ* units, parts per million (ppm), and were measured relative to residual chloroform DCCl_3_ (7.26 ppm) or DMSO-d_6_ (2.50 ppm) signals in the deuterated solvent. All ^13^C NMR spectra were reported in ppm relative to CDCl_3_ (77.16 ppm) or DMSO-d_6_ (39.52 ppm) and all spectra were obtained with ^1^H decoupling. All coupling constants *J* were reported in hertz (Hz). The following abbreviations were used to describe peak splitting patterns (s = singlet, d = doublet, t = triplet, dd = doublet of doublet, m = multiplet, and br s = broad singlet). The mass spectra were recorded on a mass spectrometer SHIMADZU GCMS-QP2010 Ultra with sample ionization by electron impact (EI) equipped with a quadrupole mass analyzer. The IR spectra were recorded using a Fourier-transform infrared spectrometer equipped with a diffuse reflection attachment. The elemental analysis was carried out on a CHNS/O analyzer. The course of the reactions was monitored by TCL on 0.25 mm silica gel plates (60F 254).

4-*tert*-Butylcalix[4]arene (1a), calix[4]arene (1b), tetramethylethylenediamine (TMEDA), *n*-buthyllithium (2.5 M solution in hexane), 2,3-dichloro-5,6-dicyano-1,4-benzoquinone (DDQ), iodocyclohexane were purchased and used as received. 5,11,17,23-Tetra-tertbutyl-25,26,27,28-tetramethoxycalix[4]arene (2a), 25,26,27,28-tetramethoxycalix[4]arene (2b),^20^ 1,3,7-triazapyrene (3)^31^ were prepared according to the literature procedures.

### General procedure for synthesis of azaheterocycles derivatives of 25,26,27,28-tetramethoxycalix[4]arene 4a, b

A Schlenk flask (100 mL) equipped with a magnetic stirrer was flame-dried under vacuum, and cooled to room temperature under an argon flow. Then TMEDA (2.25 mL, 0.00454 mol, 5.8 equiv.), THF (5 mL, dry) and *n*-BuLi (2.4 mL 2.5 M solution in hexane, 7.66 equiv.) was added, and stirred 15 minutes in acetone-liquid nitrogen bath at −20 °C. Subsequently, 2a (0.551 g, 0.000783 mol, 1 equiv.) or 2b (0.376 g, 0.000783 mol, 1 equiv.) was dissolved in THF (15 mL, dry), and the solution was cooled to −78 °C. The mixture was allowed to warm up to ambient temperature, and stirred for additional 1 h. Then 1,3,7-triazapyrene 3 (0.162 g, 0.000783 mol, 1 equiv.) was dissolved in THF (35 mL, dry) and added to the mixture, and the solution was cooled to −78 °C. The mixture again was allowed to warm up to ambient temperature and stirred for additional 1 h. After that, DDQ (0.277 g, 0.001566 mol, 2 equiv.) in THF (5 mL, dry) was added, and the reaction mixture was refluxed in oil bath for 4 h. The solvents were removed under reduced pressure, CHCl_3_ (30 mL) was added to the residual, and filtered through a short column with Al_2_O_3_ (neutral) with CHCl_3_ as eluent (100 mL). Then a solvent was removed *in vacuo*, and the desired product was purified by column chromatography on SiO_2_ with a mixture of hexane/EtOAc as an eluent.

#### 2-(1,3,7-Triazapyrene-6-yl)-5,11,17,23-tetra-*tert*-butyl-25,26,27,28-tetramethoxycalix[4]arene (4a)

White solid. Yield: 497 mg (70%), mp = 175–177 °C. *R*_f_ 0.25 (hexane/EtOAc, 8 : 2). Note: NaI (1.5 equiv.) in CD_3_CN was added to lock the conformation of calix[4]arene ^1^H NMR (DCCl_3_ + NaI in CD_3_CN, 400 MHz): *δ* 9.79 (s, 1H); 9.76 (s, 1H); 9.39 (d, 1H, *J* = 9.7 Hz); 8.60–8.58 (m, 1H); 8.435 (d, 1H, *J* = 6.0 Hz); 8.185 (d, 1H, *J* = 6.0 Hz); 7.72 (s, 1H); 7.17–7.13 (m, 8H); 4.28–4.26 (m, 2H); 4.25 (s, 6H); 4.16 (s, 6H); 3.42–3.39 (m, 4H); 1.15–1.13 (m, 36H) ppm. ^13^C{^1^H} NMR (DCCl_3_ + NaI in CD_3_CN, 100 MHz): *δ* 157.8; 157.5; 154.8; 154.0; 150.8; 150.5; 148.9; 148.8; 147.1; 136.1; 134.6; 134.5; 134.2; 133.9; 131.6; 129.2; 128.2; 126.6; 126.5; 126.2; 126.1; 122.7; 121.9; 115.5; 65.9; 64.8; 34.5; 34.2; 31.1; 31.05; 30.3; 29.9 ppm. IR (DRA): *ν* 2954, 2866, 1628, 1555, 1478, 1388, 1359, 1244, 1201, 1117, 1019, 919, 870, 798, 727, 645, 578, 557 cm^−1^. MS (EI): *m*/*z* 907 [M]^+^. Anal. calcd for C_61_H_69_N_3_O_4_: C, 80.67; H, 7.66; N, 4.63; O, 7.05. Found: C, 80.81; H, 7.88; N, 4.56.

#### 2-(1,3,7-Triazapyrene-6-yl)-25,26,27,28-tetramethoxycalix[4]arene (4b)

White solid. Yield: 320 mg (60%), mp = 165–167 °C. *R*_f_ 0.15 (hexane/EtOAc, 8 : 2). Note: the addition of NaI in CD_3_CN to this compound does not lead to the changes in the ^1^H NMR spectrum (DCCl_3_, 400 MHz): *δ* 9.87 (s, 1H); 9.67 (s, 1H); 9.10–8.80 (m, 1H); 8.62–8.58 (m, 1H); 8.32–8.20 (m, 2H); 7.49–7.32 (m, 1H); 7.09–6.94 (m, 2H); 6.85–6.54 (m, 9H); 6.46–6.13 (m, 1H); 4.48–4.36 (m, 2H); 3.96–3.65 (m, 12H); 3.54–3.45 (m, 1H); 3.29–3.19 (m, 3H) ppm. ^13^C{^1^H} NMR (DCCl_3_, 100 MHz): *δ* 159.7; 159.4; 158.3; 158.1; 158.0; 157.4; 155.2; 154.3; 147.6; 136.9; 136.7; 135.4; 135.1; 134.8; 134.0; 133.3; 132.7; 132.3; 131.6; 130.5; 130.4; 129.8; 129.4; 128.8; 128.4; 128.3; 128.0; 126.8; 122.6; 122.3; 115.8; 100.0; 61.9; 61.7; 61.1; 60.5; 41.7; 59.3; 35.8; 35.4; 30.9; 30.7; 21.2; 14.3 ppm. IR (DRA): *ν* 2935, 2845, 1760, 1683, 1597, 1497, 1424, 1349, 1204, 1085, 969, 851, 722, 612, 548 cm^−1^. MS (EI): *m*/*z* 683 [M]^+^. Anal. calcd for C_45_H_37_N_3_O_4_: C, 79.04; H, 5.45; N, 6.15; O, 9.36. Found: C, 79.15; H, 5.71; N, 5.92.

### General procedure for synthesis of azaheterocycles derivatives of 25,26,27,28-tetrahydroxycalix[4]arene 5a, b

In a round-bottom flask (50 mL) equipped with a magnetic stirrer, 4a (0.362 g, 0.0004 mol, 1 equiv.) or 4b (0.273 g, 0.0004 mol, 1 equiv.) was placed in DMF (25 mL, dry). Subsequently, iodocyclohexane (2.55 mL, 0.0178 mol, 44.5 equiv.) was added, and the reaction mixture was refluxed in oil bath for 6 hours. Then H_2_O (200 mL, distilled) was added, and the reaction mixture was extracted CHCl_3_ (4 × 70 mL). The organic phase was washed with saturated Na_2_S_2_O_3_ (2 × 100 mL), dried over Na_2_SO_4_ and concentrated *in vacuo*. The desired product was purified by column chromatography with the appropriated eluent (for 5a: benzene/methanol 100/1 mixture; for 5b: CHCl_3_).

#### 2-(1,3,7-Triazapyrene-6-yl)-5,11,17,23-tetra-*tert*-butyl-25,26,27,28-tetrahydroxycalix[4]arene (5a)

Yellow solid. Yield: 221 mg (65%), mp = 172–174 °C. *R*_f_ 0.4 (benzene/MeOH, 100 : 1). ^1^H NMR (DCCl_3_, 400 MHz): *δ* 11.78 (br s, 4H); 9.90 (s, 1H); 9.65 (s, 1H); 8.98 (d, 1H, *J* = 4 Hz); 8.655 (d, 1H, *J* = 12 Hz); 8.29 (d, 1H, *J* = 4 Hz); 8.255 (d, 1H, *J* = 12 Hz); 7.45–7.35 (m, 2H); 7.13–7.06 (m, 6H); 6.53–6.40 (m, 1H); 4.37–4.15 (m, 3H); 3.56–3.35 (m, 3H); 1.25 (s, 36H) ppm. ^13^C{^1^H} NMR (DCCl_3_, 100 MHz): *δ* 158.4; 155.5; 154.3; 149.3; 147.8; 143.8; 134.5; 132.5; 130.0; 129.7; 129.1; 128.8; 128.3; 127.8; 127.4; 126.1; 125.9; 122.2; 115.6; 34.2; 34.1; 33.3; 33.2; 31.6 ppm. IR (DRA): *ν* 3888, 3749, 3643, 3542, 3426, 3281, 2951, 1735, 1629, 1594, 1480, 1390, 1359, 1295, 1198, 987, 872, 815, 704, 589, 527 cm^−1^. MS (EI): *m*/*z* 852 [M]^+^. Anal. calcd for C_57_H_61_N_3_O_4_: C, 80.34; H, 7.22; N, 4.93; O, 7.51. Found: C, 80.32; H, 7.58; N, 4.75.

#### 2-(1,3,7-Triazapyrene-6-yl)-25,26,27,28-tetrahydroxycalix[4]arene (5b)

Yellow solid. Yield: 150 mg (60%), mp = 168–170 °C. *R*_f_ 0.45 (CHCl_3_, 100%). ^1^H NMR (DCCl_3_, 400 MHz): *δ* 11.58 (br s, 4H); 9.88 (s, 1H); 9.62 (s, 1H); 8.88 (d, *J* = 9.5 Hz, 1H); 8.61 (d, *J* = 9.2 Hz, 1H); 8.27–8.21 (m, 2H); 7.56–7.34 (m, 2H); 7.11–7.02 (m, 6H); 6.82–6.70 (m, 4H); 6.64–6.42 (m, 1H); 4.32–4.00 (m, 3H); 3.73–3.38 (m, 3H); ppm. ^13^C{^1^H} NMR (DCCl_3_, 100 MHz): *δ* 158.4; 155.4; 154.2; 151.4; 149.6; 143.6; 134.3; 132.2; 130.3; 130.1; 129.2; 129.0; 128.8; 128.7; 128.5; 127.4; 123.4; 122.1; 121.9; 121.7; 115.4; 32.3; 32.0 ppm. IR (DRA): *ν* 3841, 3747, 3655, 3566, 3436, 3192, 3039, 2945, 1667, 1628, 1591, 1501, 1448, 1385, 1251, 1083, 891, 788, 750, 560, 515 cm^−1^. MS (EI): *m*/*z* 627 [M]^+^. Anal. calcd for C_41_H_29_N_3_O_4_: C, 78.45; H, 4.66; N, 6.69; O, 10.20. Found: C, 78.37; H, 4.74; N, 6.43.

## Conflicts of interest

There are no conflicts to declare.

## Supplementary Material

RA-011-D0RA10605D-s001
