# Supplementary material for: Meso-functionalization of calix[4]arene with 1,3,7-triazapyrene in the design of novel fluorophores with the dual target detection of Al3+ and Fe3+ cations
Source: RSC Adv. 2021 Feb 4;11(11):6407–14. doi: 10.1039/d0ra10605d (PMC8694849; doi:10.1039/d0ra10605d)
Supplement: RA-011-D0RA10605D-s001 [file RA-011-D0RA10605D-s001.pdf]

Electronic Supplementary Material (ESI) for RSC Advances.
This journal is © The Royal Society of Chemistry 2021

Electronic Supplementary Information (ESI)

Meso-functionalization of calix[4]arene with 1,3,7-triazapyrene in the design of novel fluorophores with the dual target detection of Al^{3+} and Fe^{3+} cations

Timofey D. Moseev,[a] Igor A. Lavrinchenko,[a] Mikhail V. Varaksin,[a,b]*
Diana Yu. Pobedinskaya,[c] Oleg P. Demidov,[c] Ivan V. Borovlev,[c]
Valery N. Charushin,[a,b] and Oleg N. Chupakhin[a,b]*

[a] *Ural Federal University, 19 Mira Str., 620002 Ekaterinburg, Russia*
[b] *Institute of Organic Synthesis, Ural Branch of the Russian Academy of Sciences, 22 S. Kovalevskaya Str., 620990 Ekaterinburg, Russia*
[c] *North-Caucasus Federal University, 355009 Stavropol, Russia*

E-mail: m.v.varaksin@urfu.ru

E-mail: chupakhin@ios.uran.ru

TABLE OF CONTENTS

1. **Copies of ^{1}H, ^{13}C NMR spectra for 2-(1,3,7-triazapyrene-6-yl)-5,11,17,23-tetra-*tert*-butyl-25,26,27,28-tetramethoxycalix[4]arene (4a)** **S2**
2. **Copies of ^{1}H, ^{13}C NMR spectra for 2-(1,3,7-triazapyrene-6-yl)-25,26,27,28-tetramethoxycalix[4]arene (4b)** **S6**
3. **Copies of ^{1}H, ^{13}C NMR spectra for 2-(1,3,7-triazapyrene-6-yl)-5,11,17,23-tetra-*tert*-butyl-25,26,27,28-tetrahydroxycalix[4]arene (5a)** **S10**
4. **Copies of ^{1}H, ^{13}C NMR spectra for 2-(1,3,7-triazapyrene-6-yl)-25,26,27,28-tetrahydroxycalix[4]arene (5b)** **S13**
5. **Photophysical studies** **S20**
   - **5.1. Absorbance spectra** **S20**
   - **5.2. Emission spectra** **S23**
   - **5.3. Emission spectra in the presence of various metal ions** **S25**
6. **Determining the stoichiometry of ligand: metal complex** **S27**
7. **Binding constants measurement** **S28**
8. **Emission spectra depending on pH** **S31**
9. **References** **S32**

1. **Copies of ^{1}H, ^{13}C NMR spectra for 2-(1,3,7-triazapyrene-6-yl)-5,11,17,23-tetra-*tert*-butyl-25,26,27,28-tetramethoxycalix[4]arene (4a)**

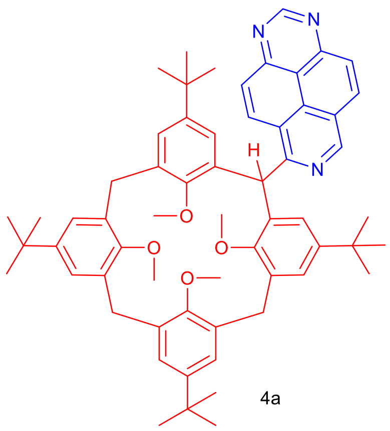

4a

Spectrum S1. ^{1}H NMR (400 MHz, $CDCl_3$) of **4a**

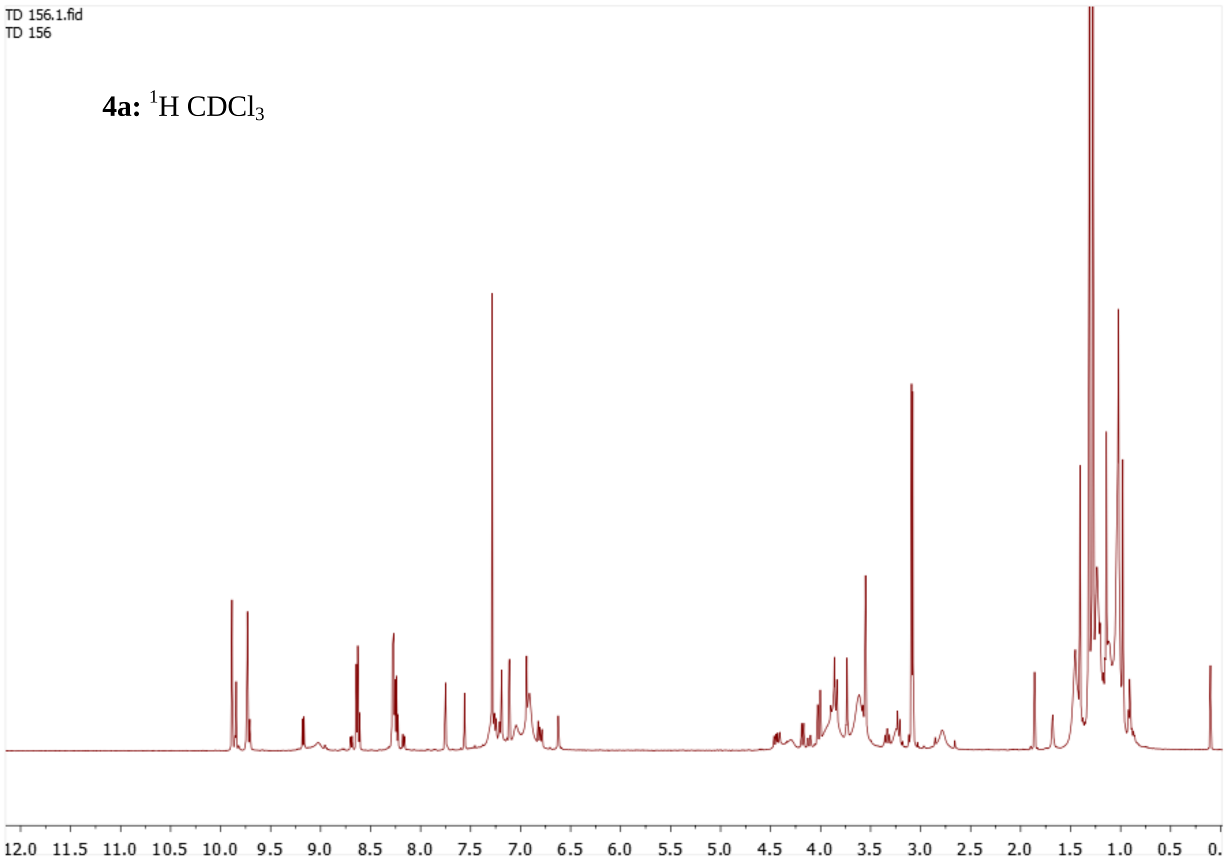

Spectrum S2. ^{1}H NMR (400 MHz, $CDCl_3$) of **4a** with addition of NaI

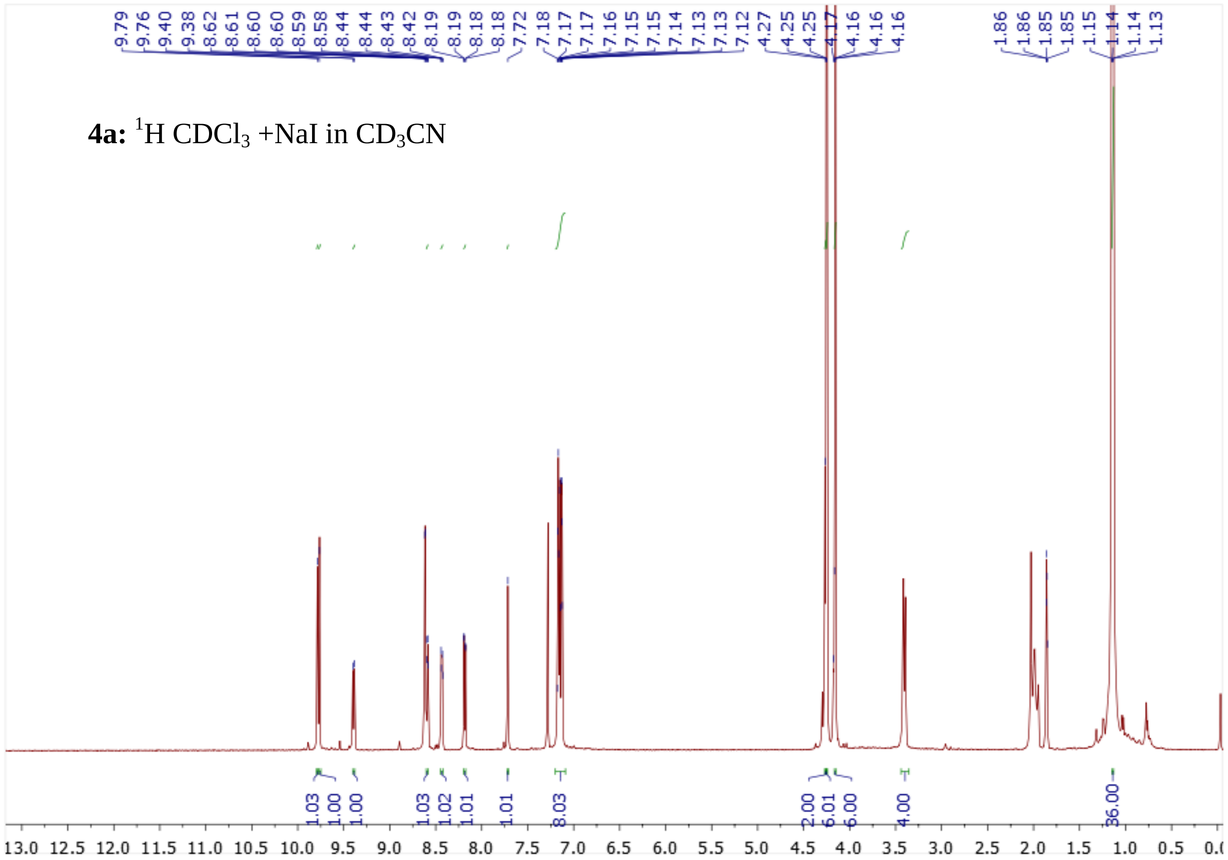

Spectrum S3. $^{13}C\{^{1}H\}$ NMR (100 MHz, $CDCl_3$) of **4a**

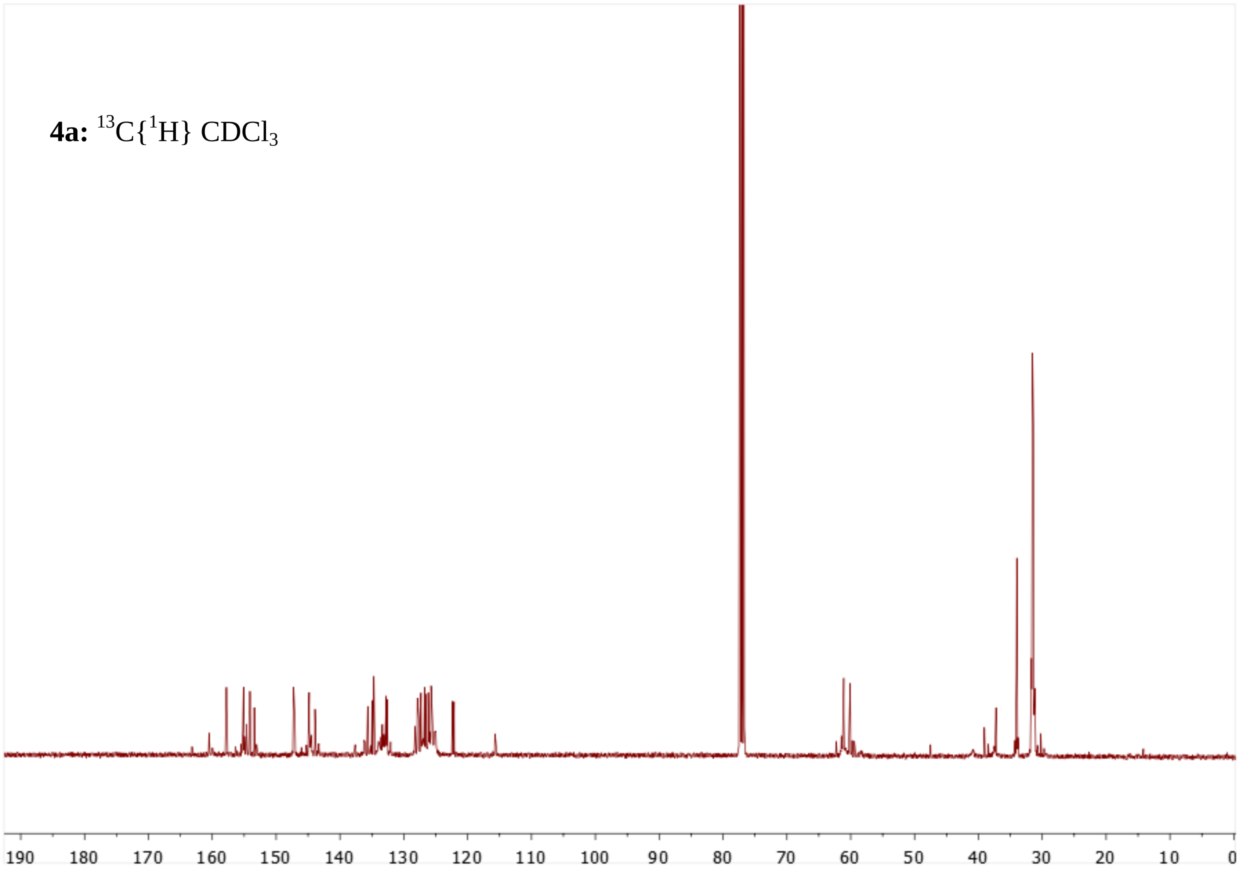

Spectrum S4. $^{13}C\{^1H\}$ NMR (100 MHz, $CDCl_3$) of **4a** with addition of NaI

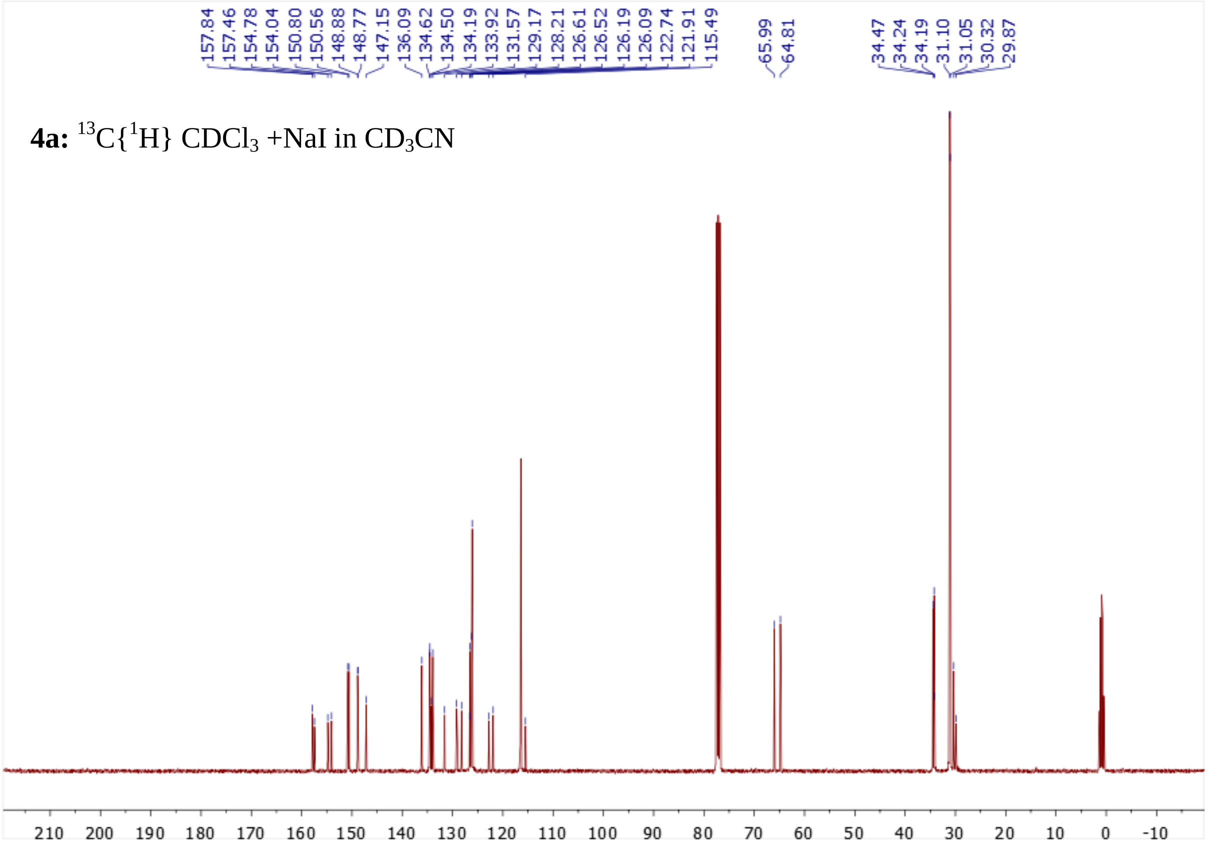

Spectrum S5. ^1H -^{13}C HSQC NMR ($CDCl_3$) of **4a** with addition of NaI

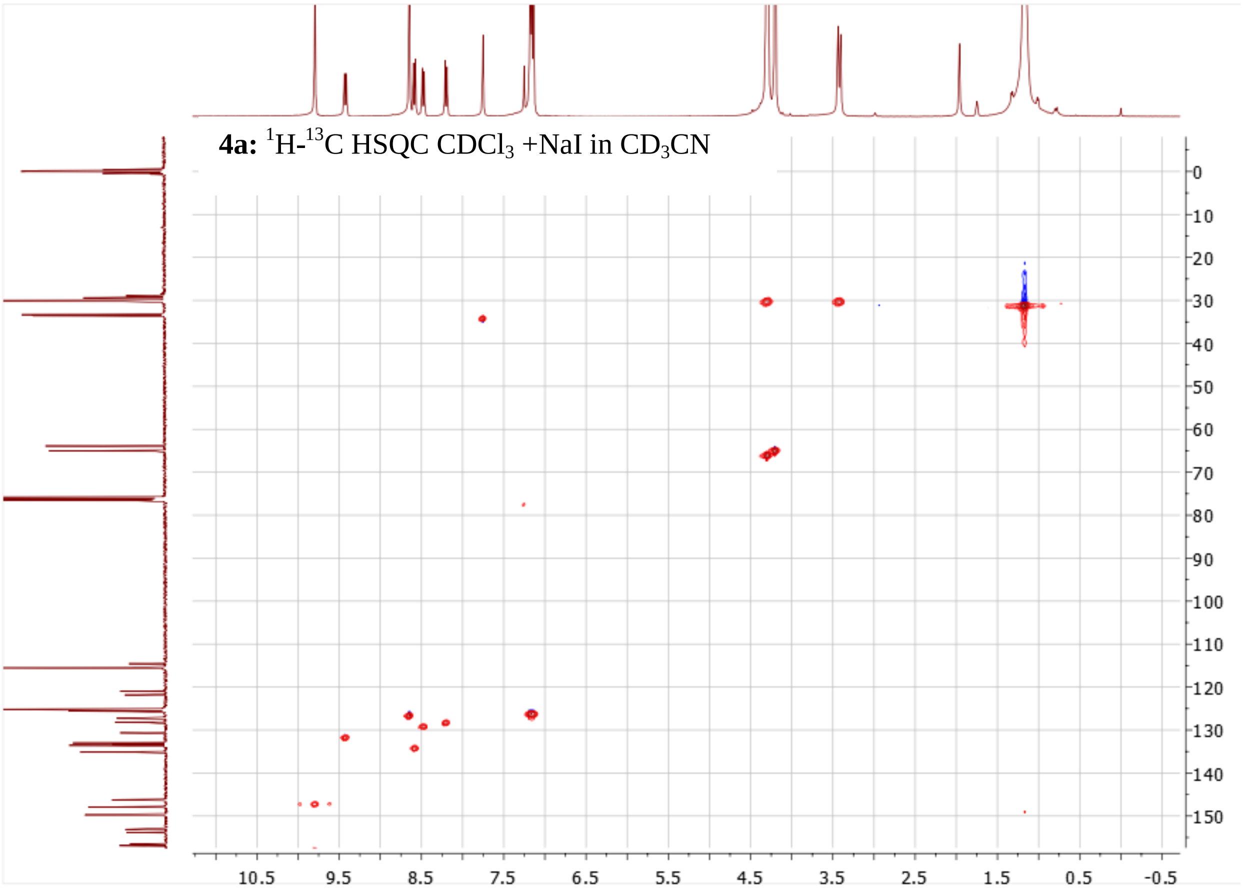

Spectrum S6. ^{1}H -^{13}C HMBC NMR ($CDCl_3$) of **4a** with addition of NaI

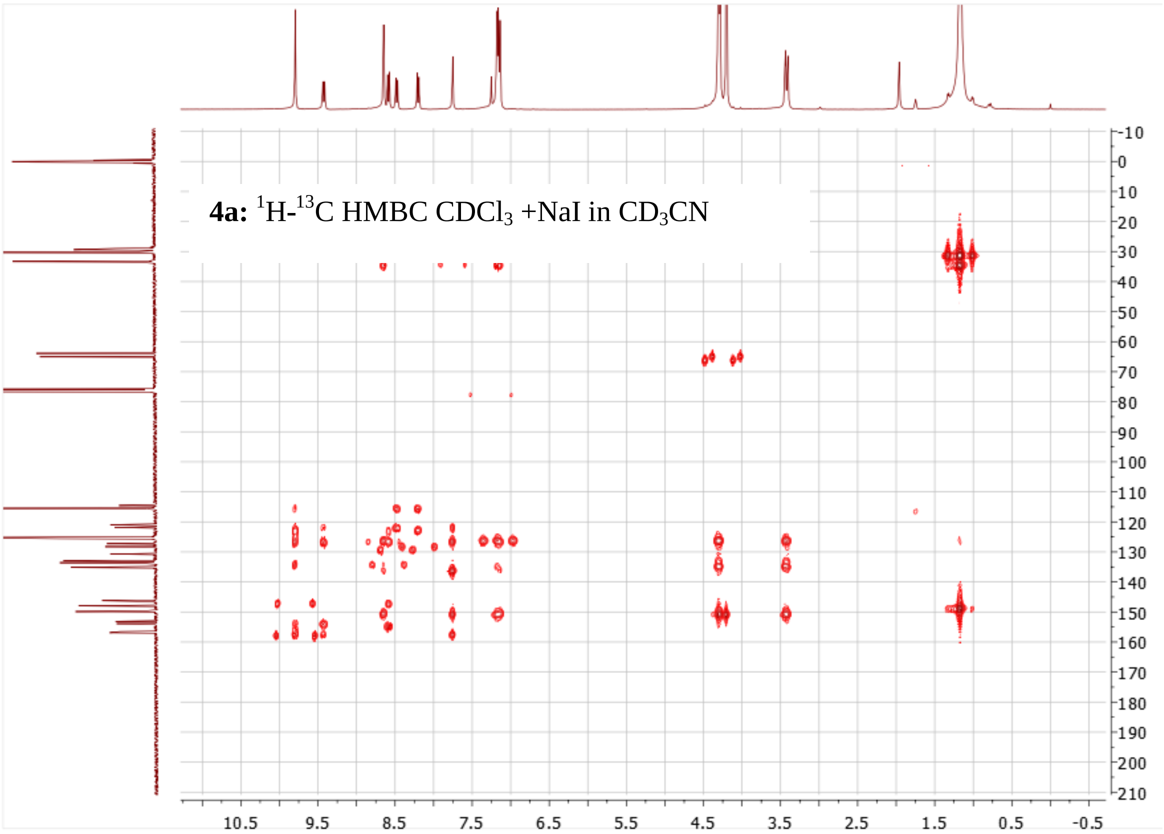

Spectrum S7. ^{1}H -^{1}H COSY NMR ($CDCl_3$) of **4a** with addition of NaI

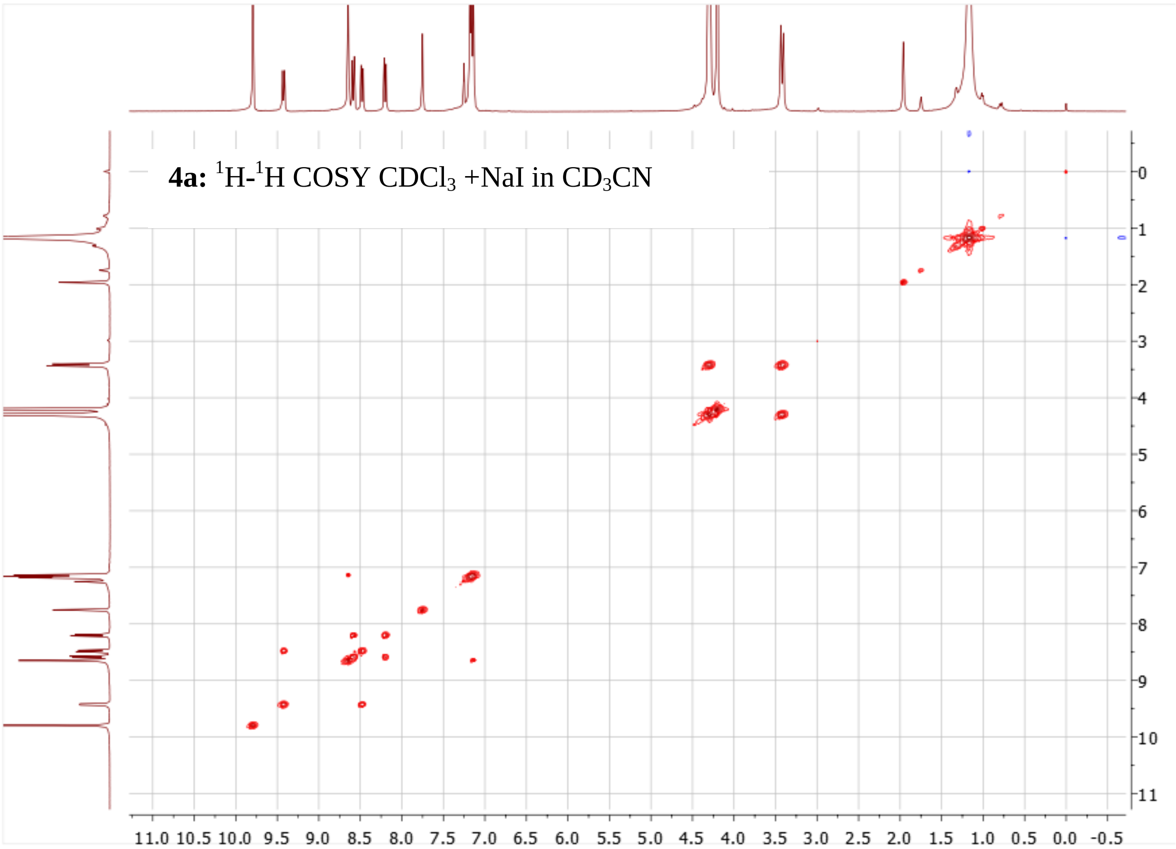

2. Copies of ^{1}H, ^{13}C NMR spectra for 2-(1,3,7-triazapyrene-6-yl)-25,26,27,28-tetramethoxycalix[4]arene (4b)

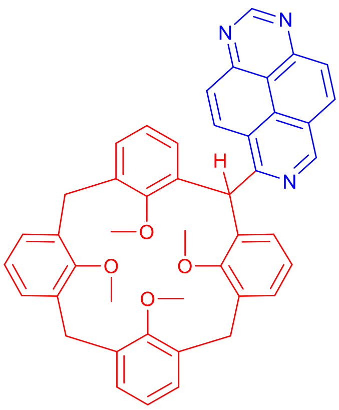

4b

Spectrum S8. ^{1}H NMR (400 MHz, $CDCl_3$) of **4b**

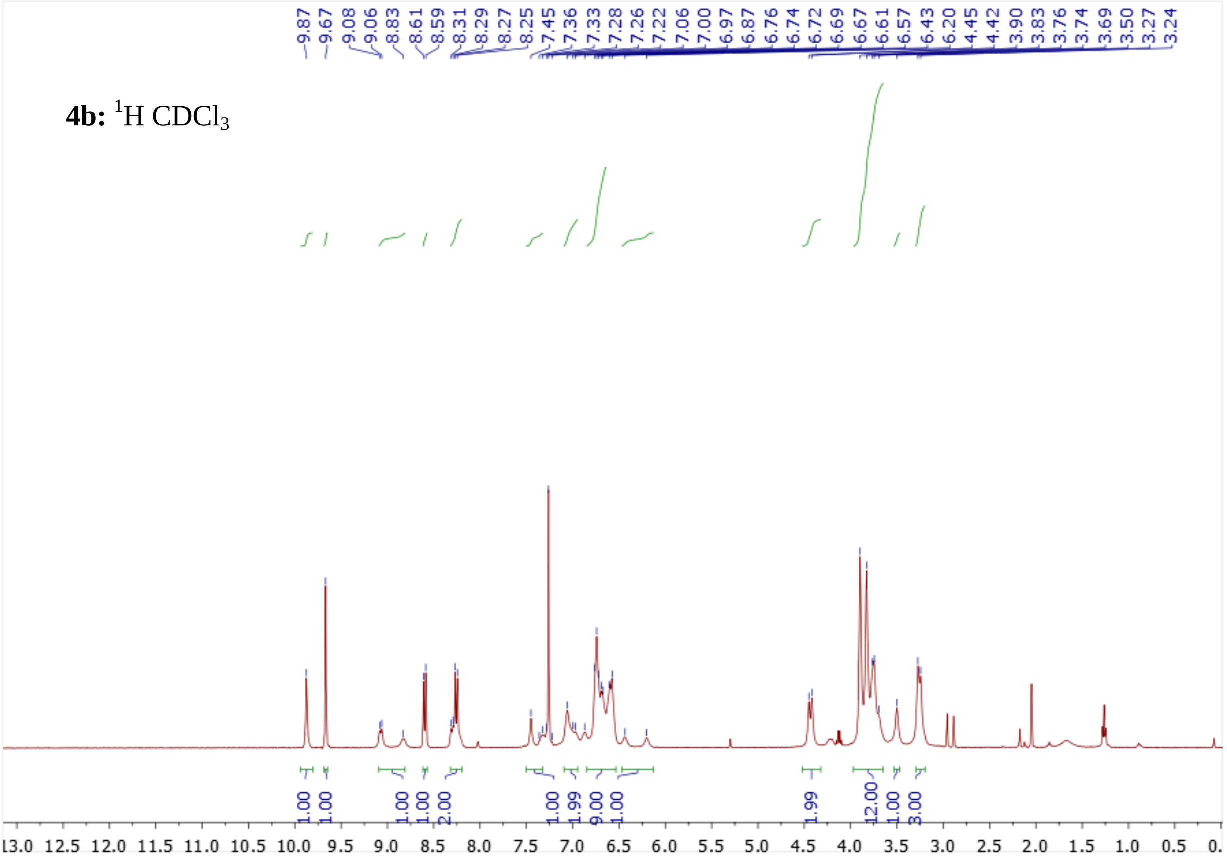

Spectrum S9. ^{1}H NMR (400 MHz, $CDCl_3$) of **4b** with addition of NaI

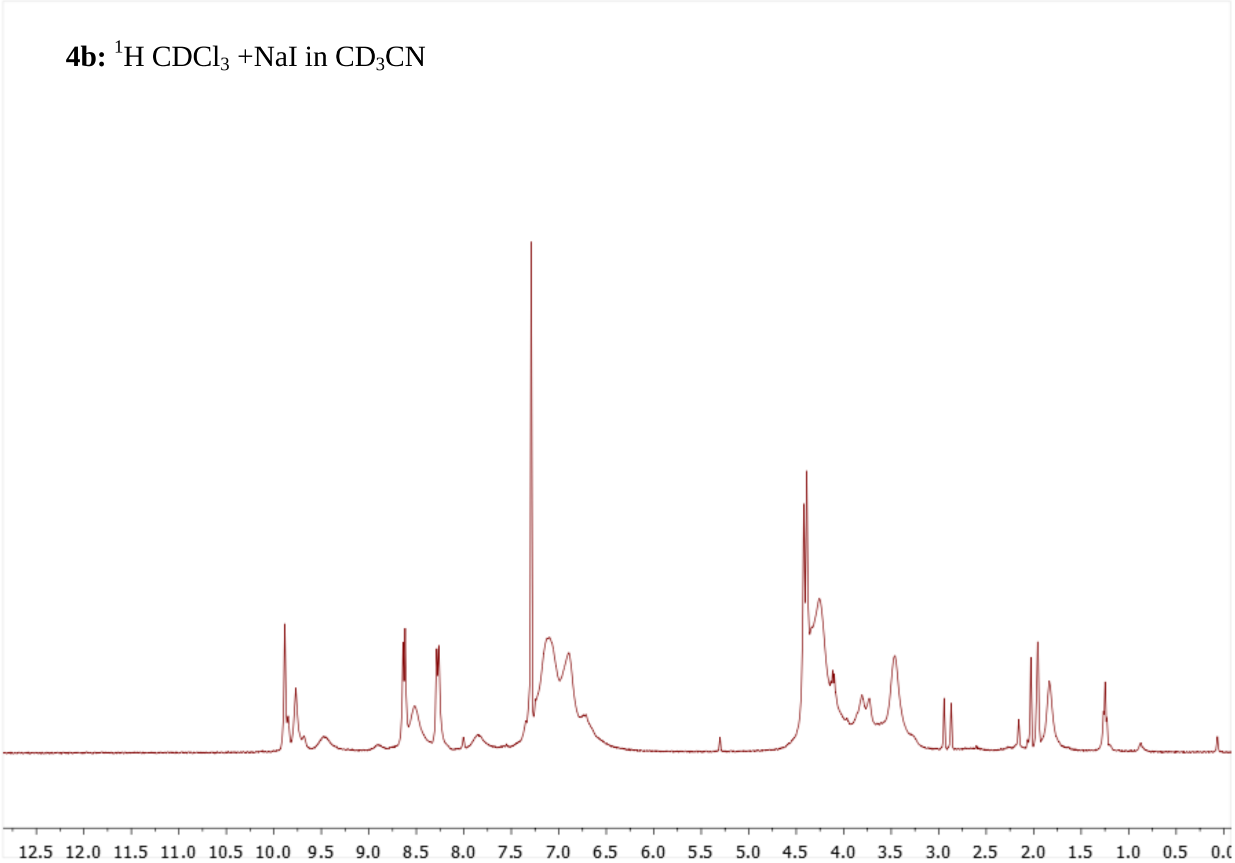

Spectrum S10. $^{13}C\{^1H\}$ NMR (100 MHz, $CDCl_3$) of **4b**

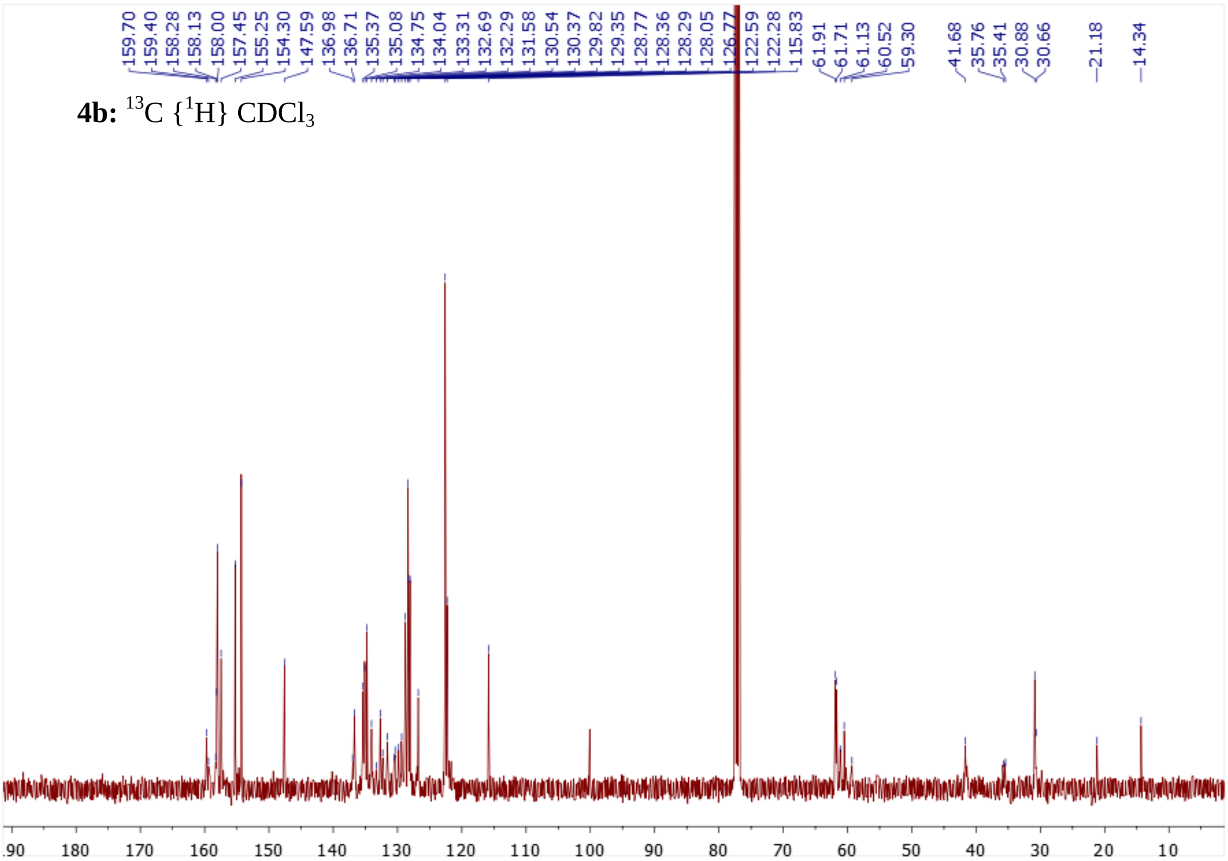

Spectrum S11. ^{1}H -^{13}C HSQC NMR ($CDCl_3$) of **4b**

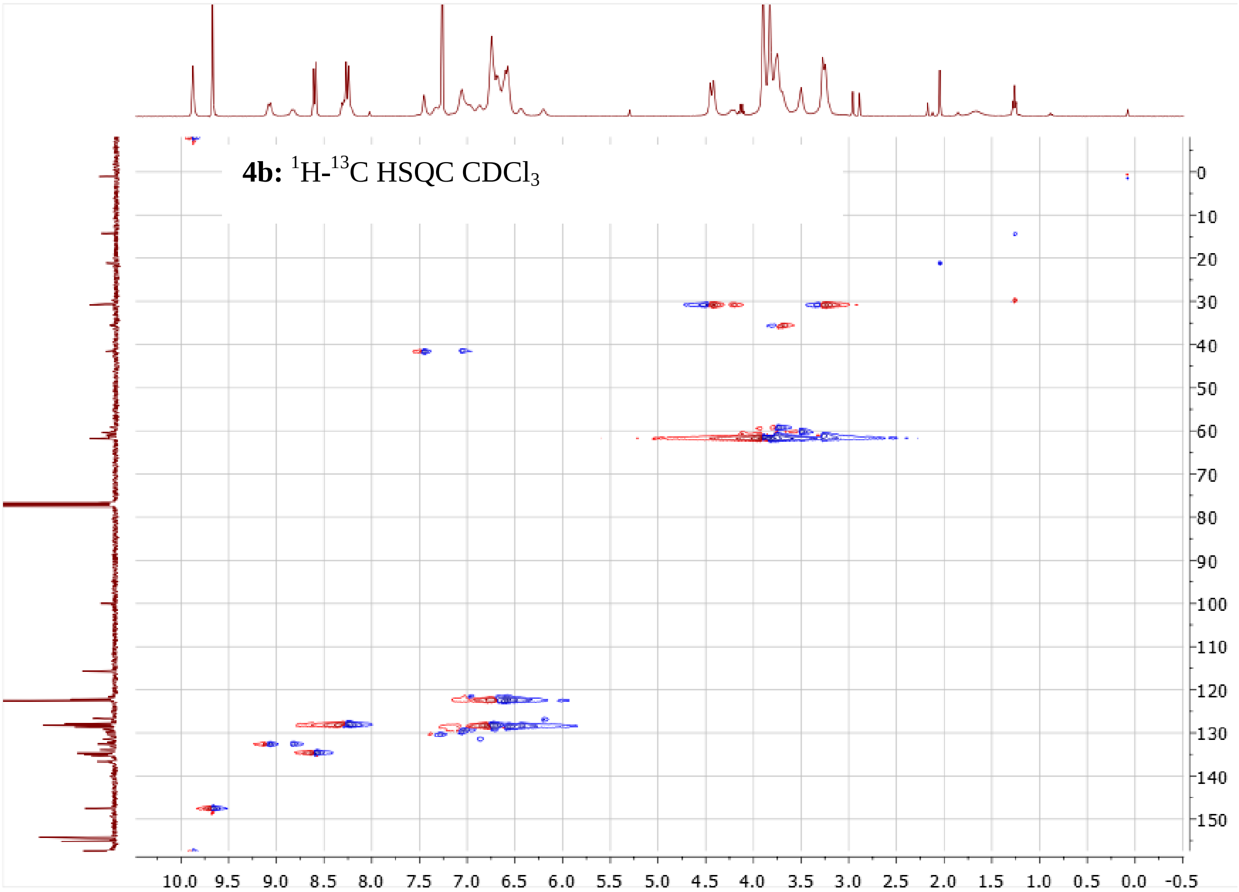

Spectrum S12. ^{1}H -^{13}C HMBC NMR ($CDCl_3$) of **4b**

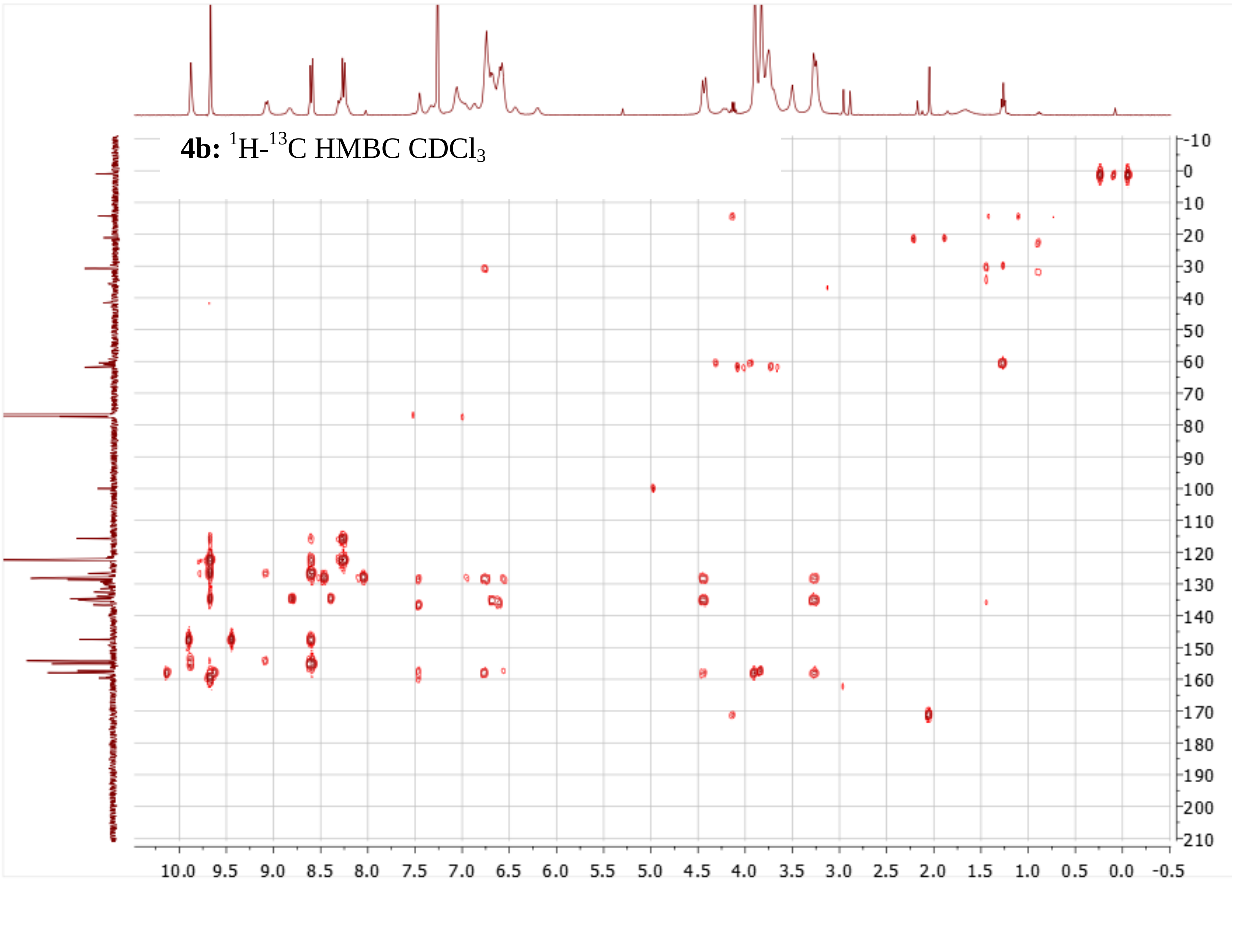

Spectrum S13. ^{1}H -^{1}H COSY NMR ($CDCl_3$) of **4b**

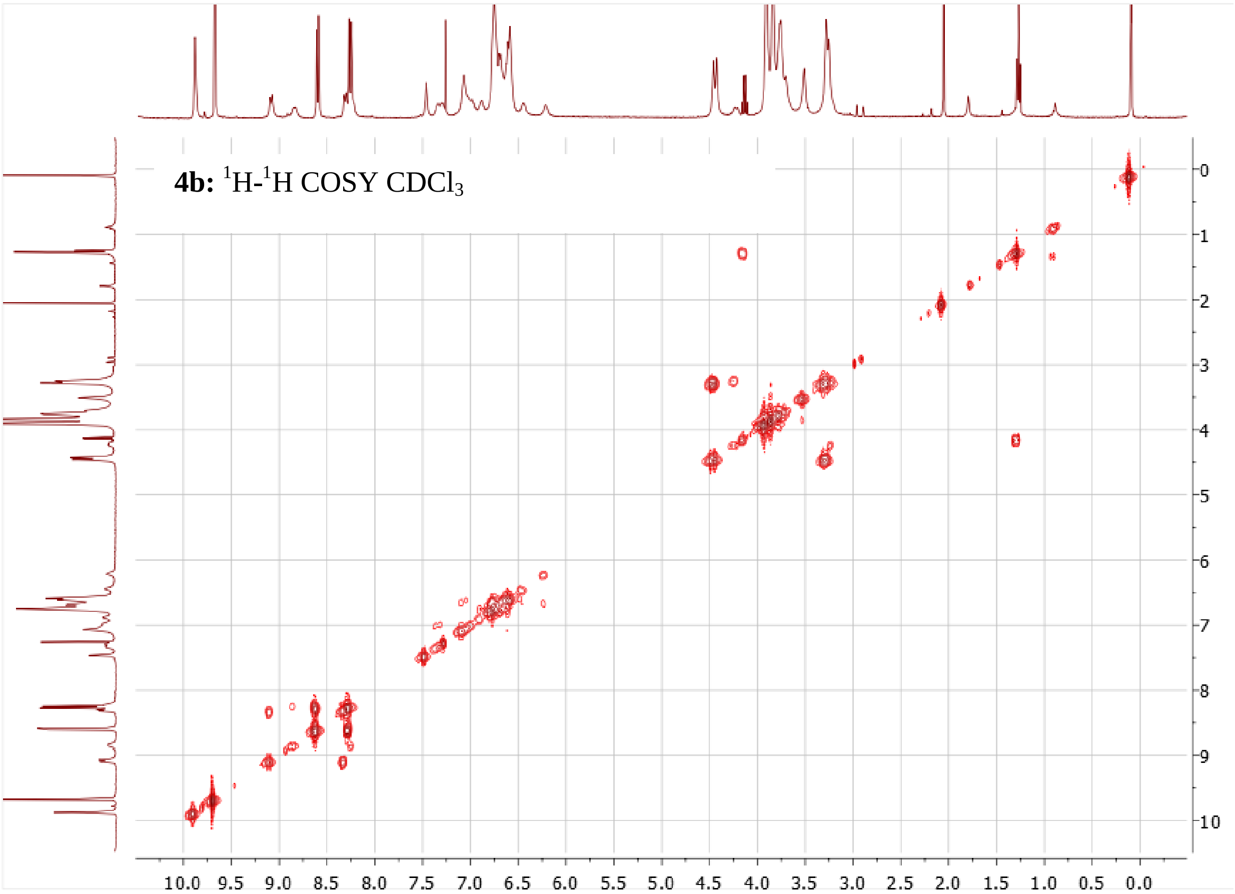

3. Copies of ^{1}H, ^{13}C NMR spectra for 2-(1,3,7-triazapyrene-6-yl)-5,11,17,23-tetra-*tert*-butyl-25,26,27,28-tetrahydroxycalix[4]arene (5a)

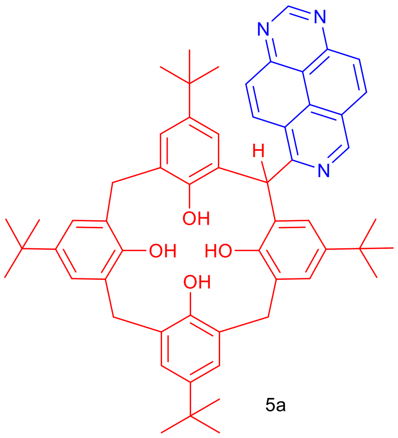

Spectrum S14. ^{1}H NMR (400 MHz, $CDCl_3$) of **5a**

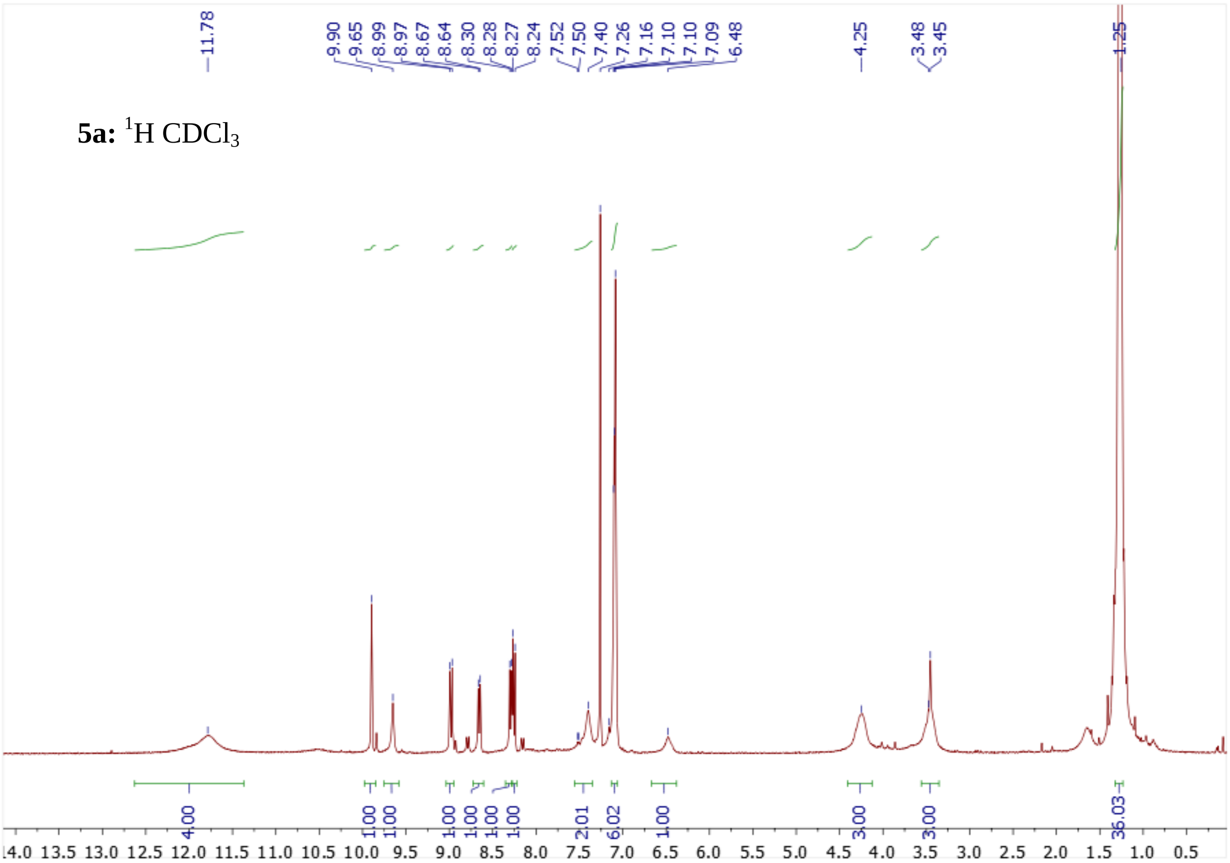

Spectrum S15. $^{13}C\{^1H\}$ NMR (100 MHz, $CDCl_3$) of **5a**

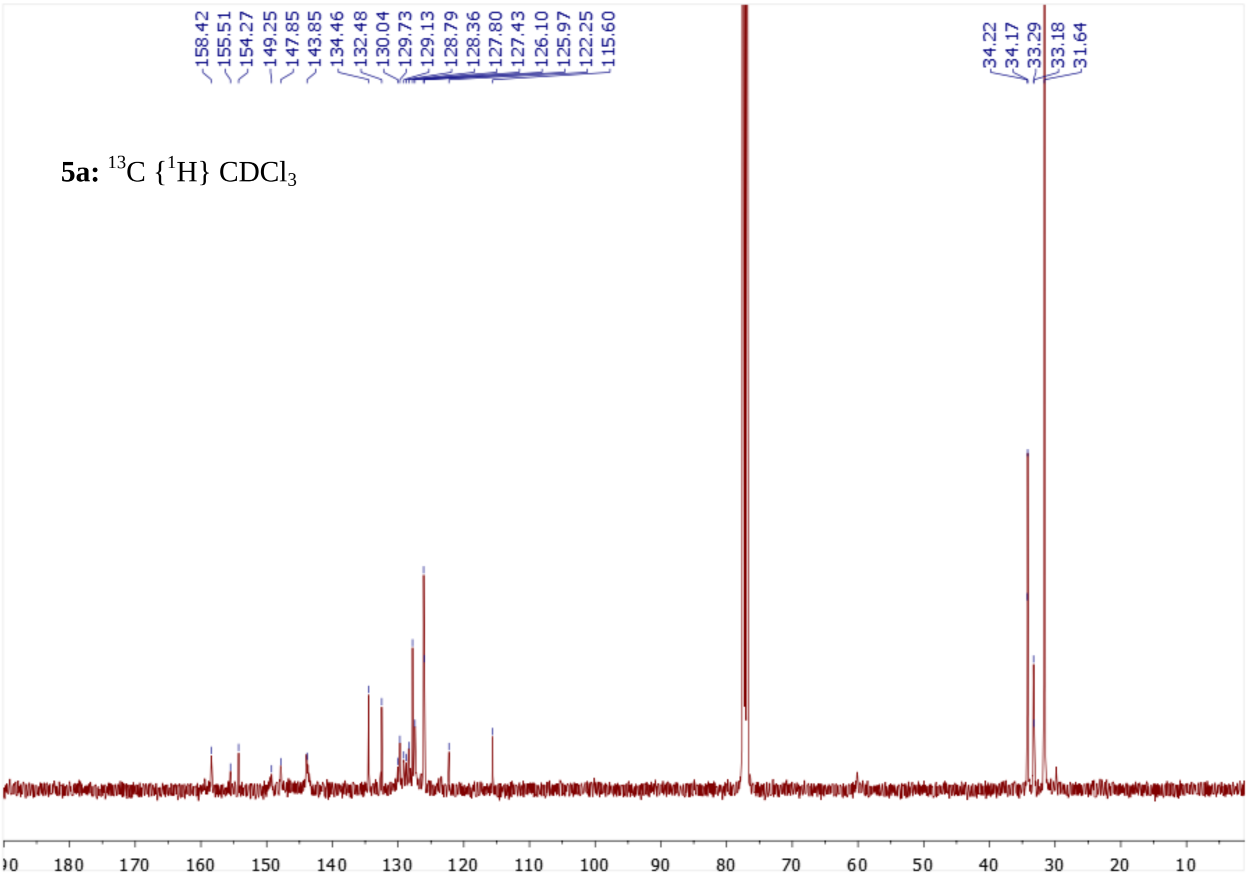

Spectrum S16. ^1H-^{13}C HSQC NMR ($CDCl_3$) of **5a**

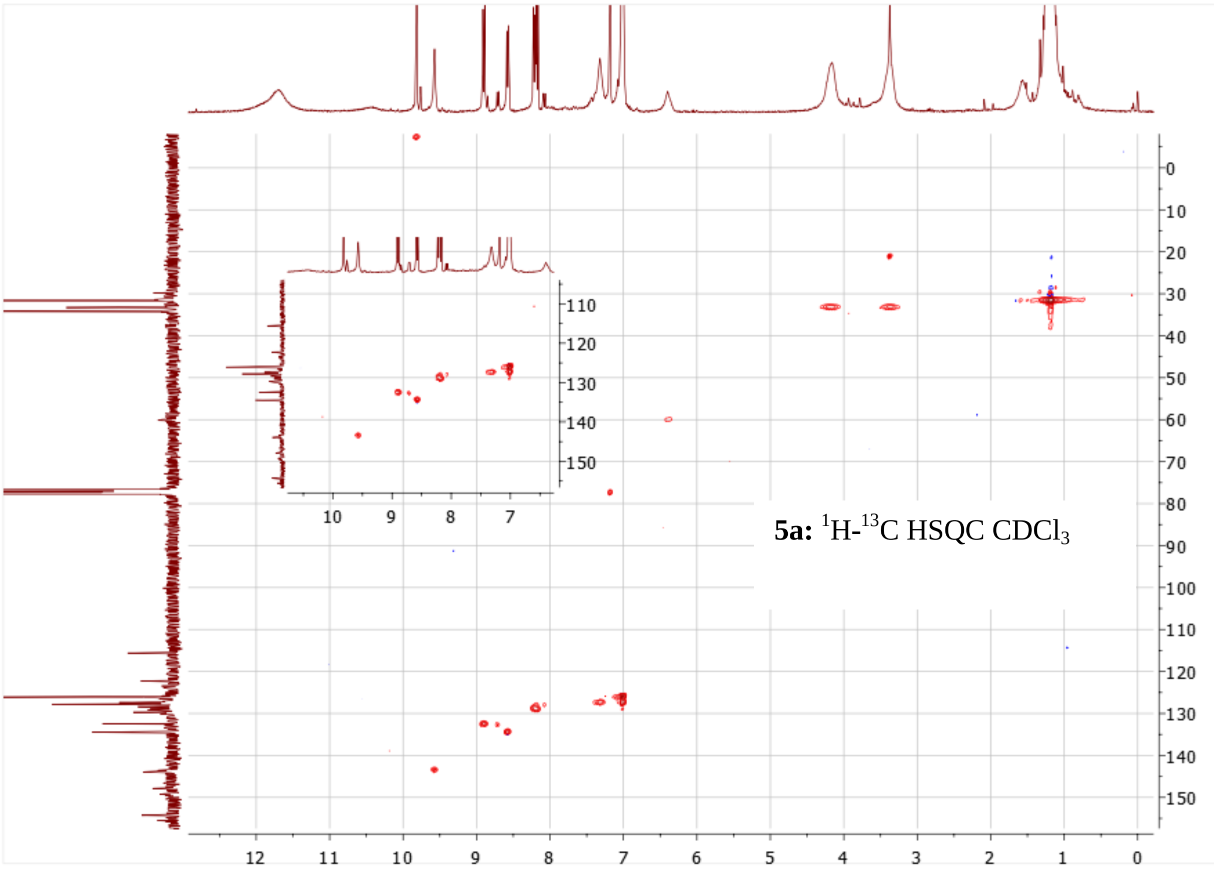

Spectrum S17. ^{1}H-^{13}C HMBC NMR ($CDCl_3$) of **5a**

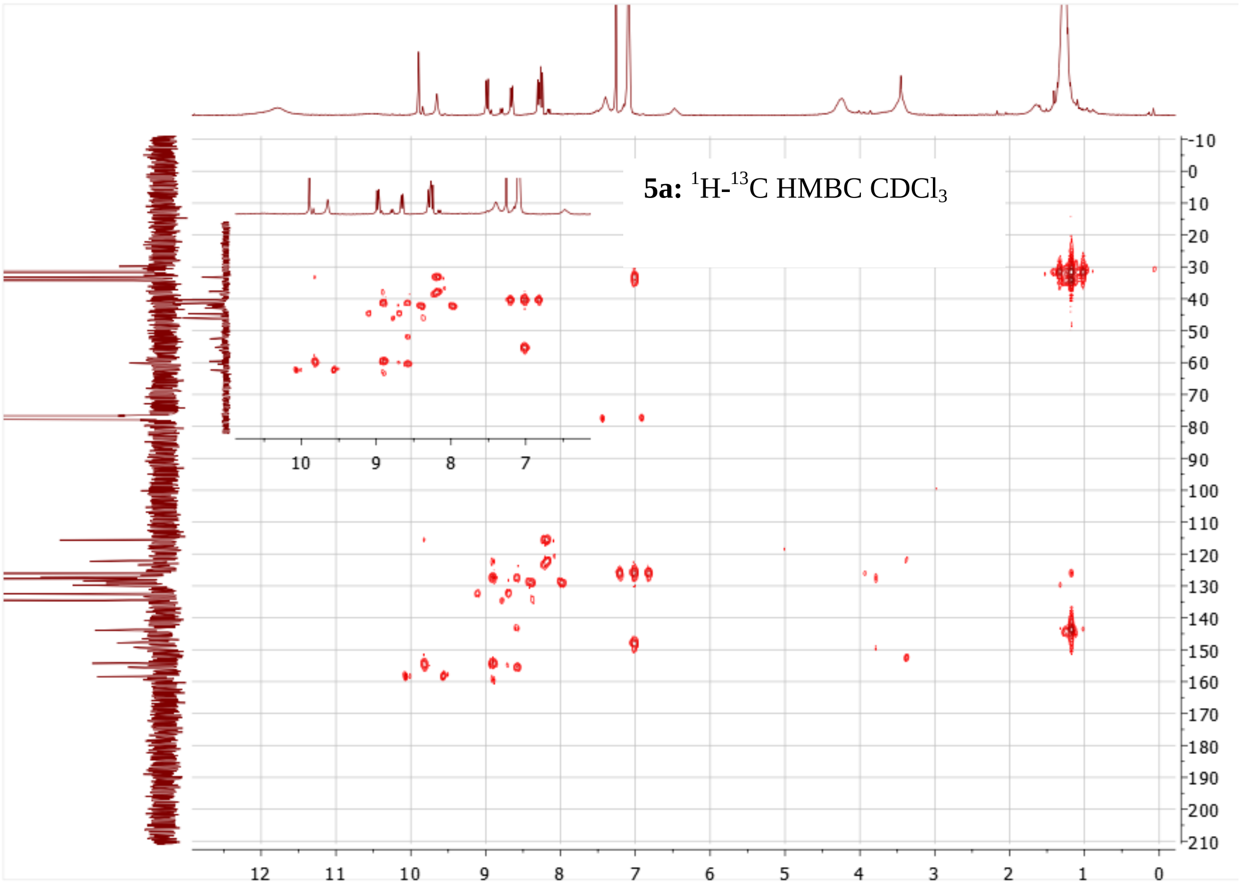

Spectrum S18. ^{1}H-^{1}H COSY NMR ($CDCl_3$) of **5a**

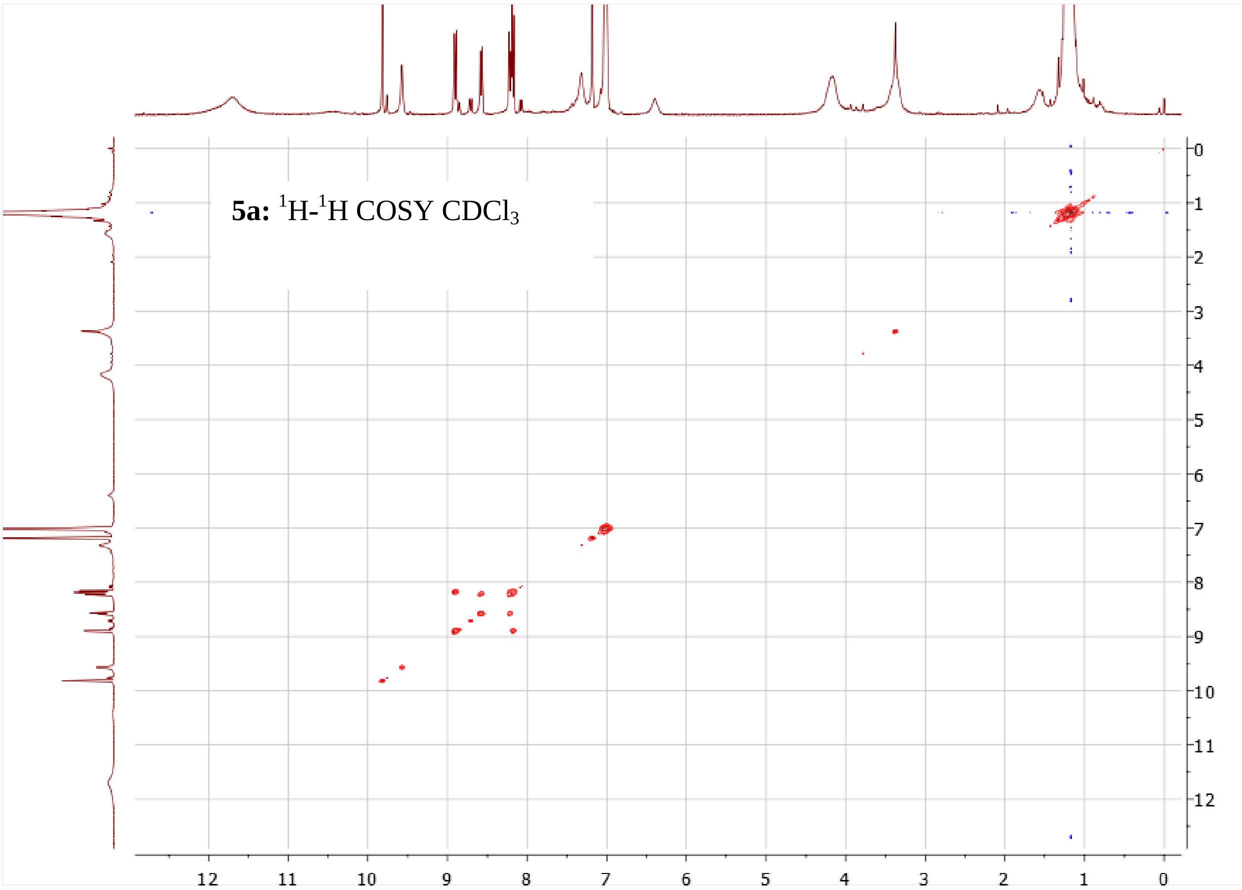

4. Copies of ^{1}H, ^{13}C NMR spectra for 2-(1,3,7-triazapyrene-6-yl)-25,26,27,28-tetrahydroxycalix[4]arene (5b)

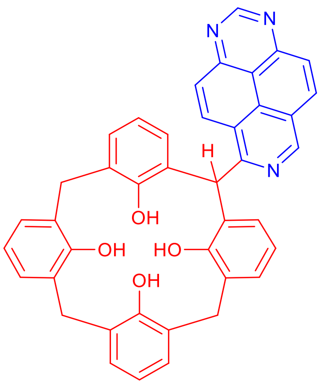

5b

Spectrum S19. ^{1}H NMR (400 MHz, $CDCl_3$) of **5b**

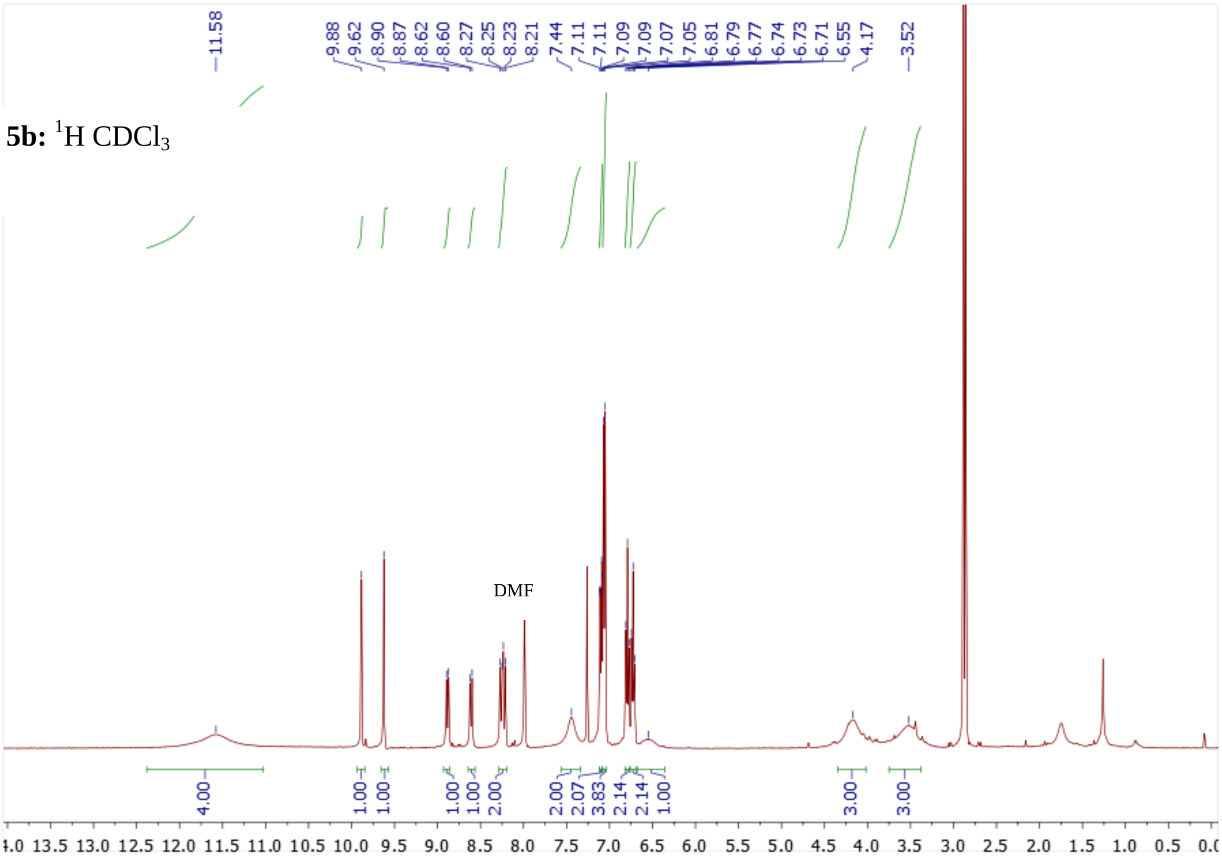

Spectrum S20. ^{13}C {^{1}H} NMR (100 MHz, $CDCl_3$) of **5b**

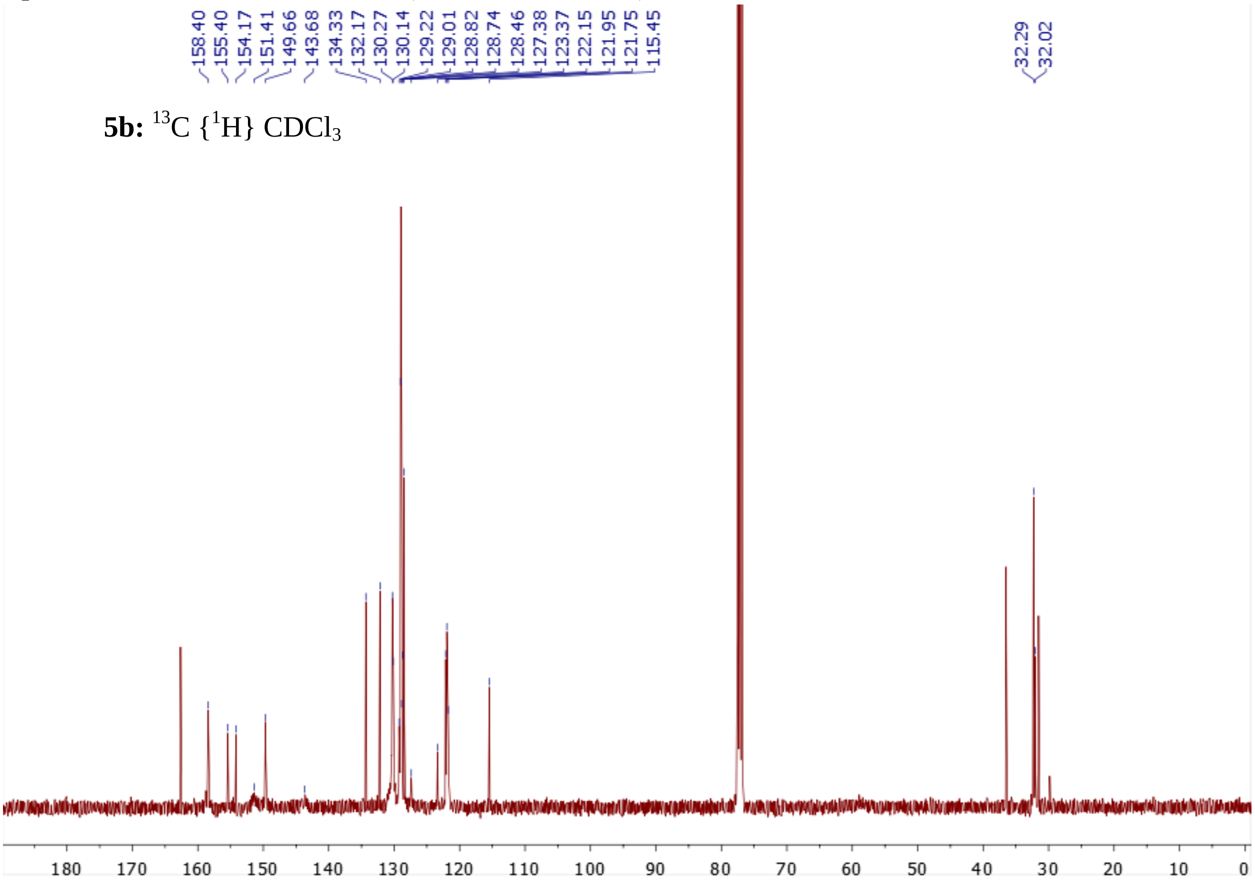

Spectrum S21. ^{1}H-^{13}C HSQC NMR ($CDCl_3$) of **5b**

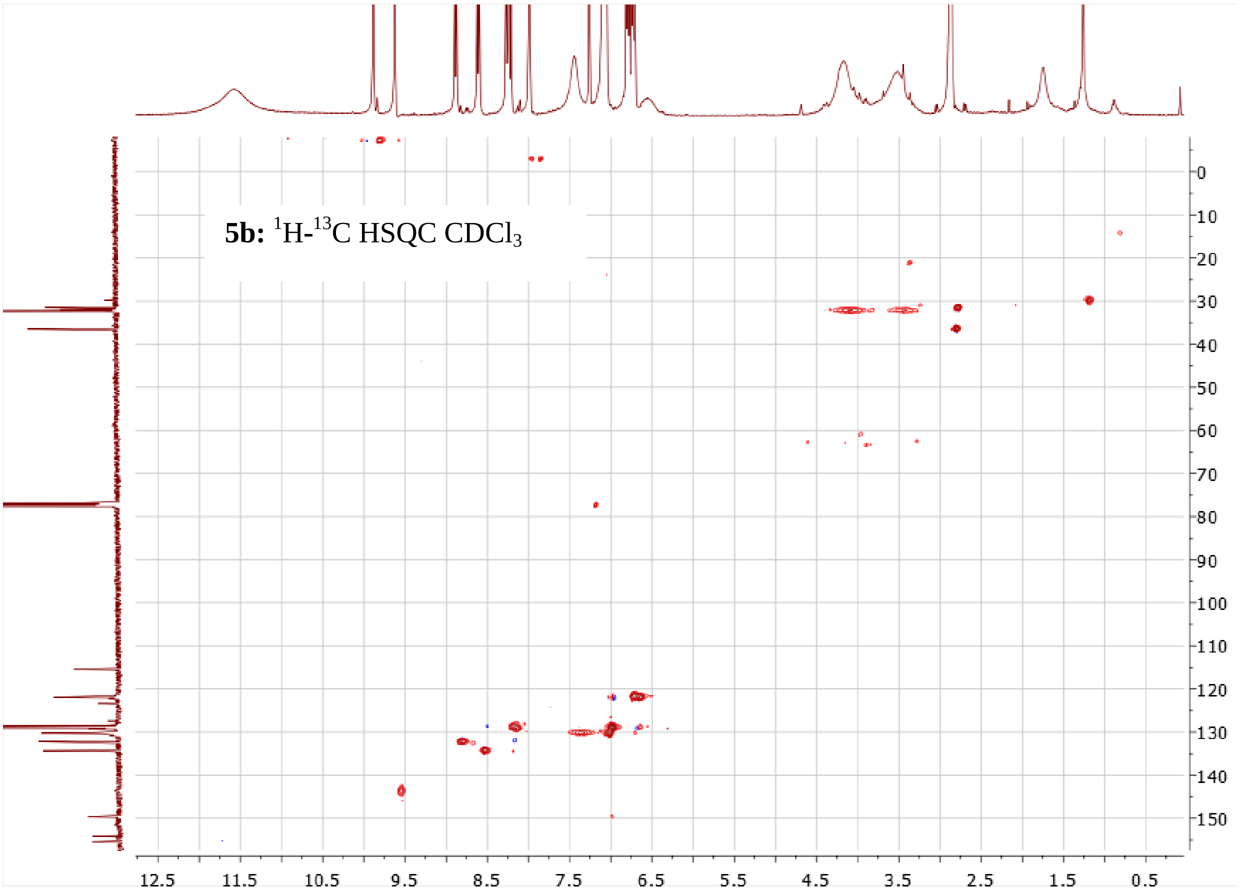

Spectrum S22. ^{1}H-^{13}C HMBC NMR ($CDCl_3$) of **5b**

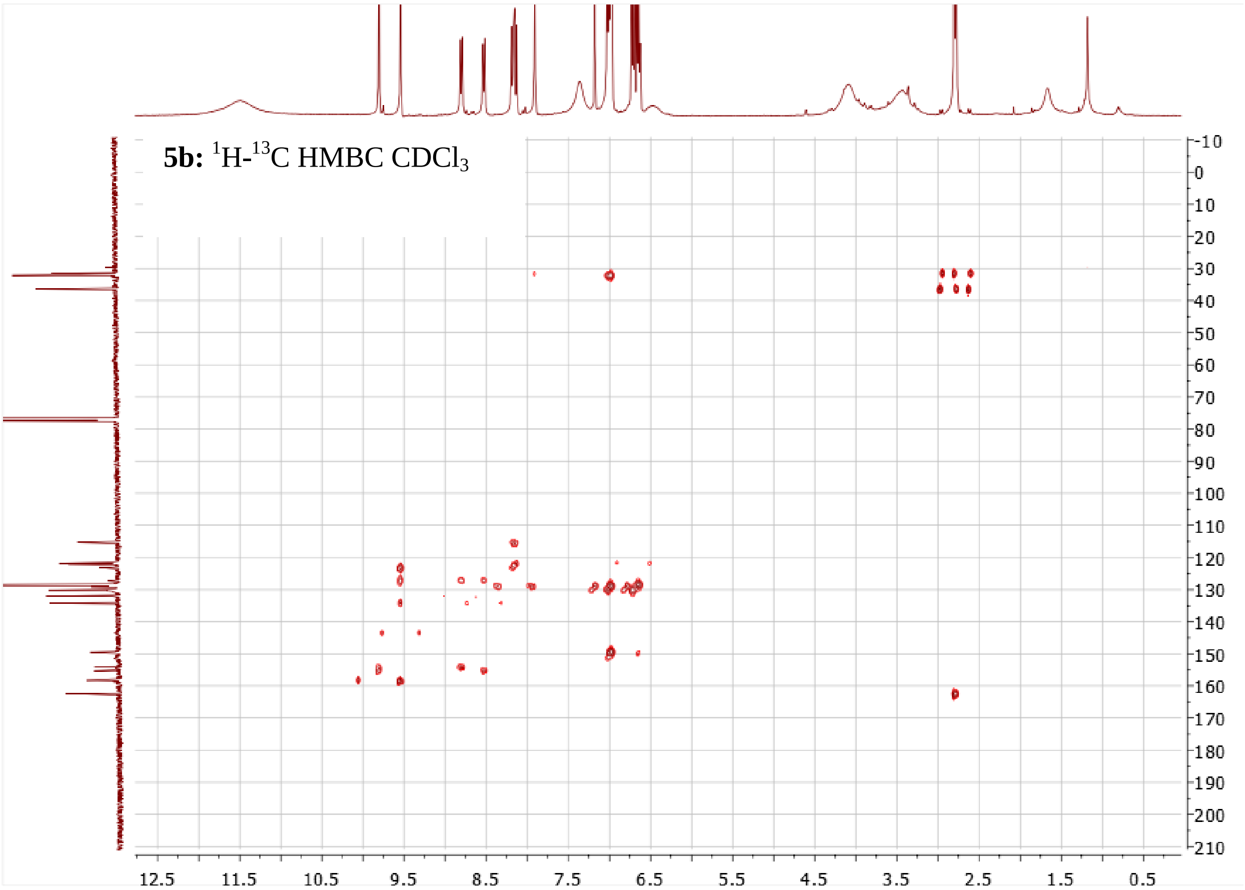

Spectrum S23. ^{1}H-^{1}H COSY NMR ($CDCl_3$) of **5b**

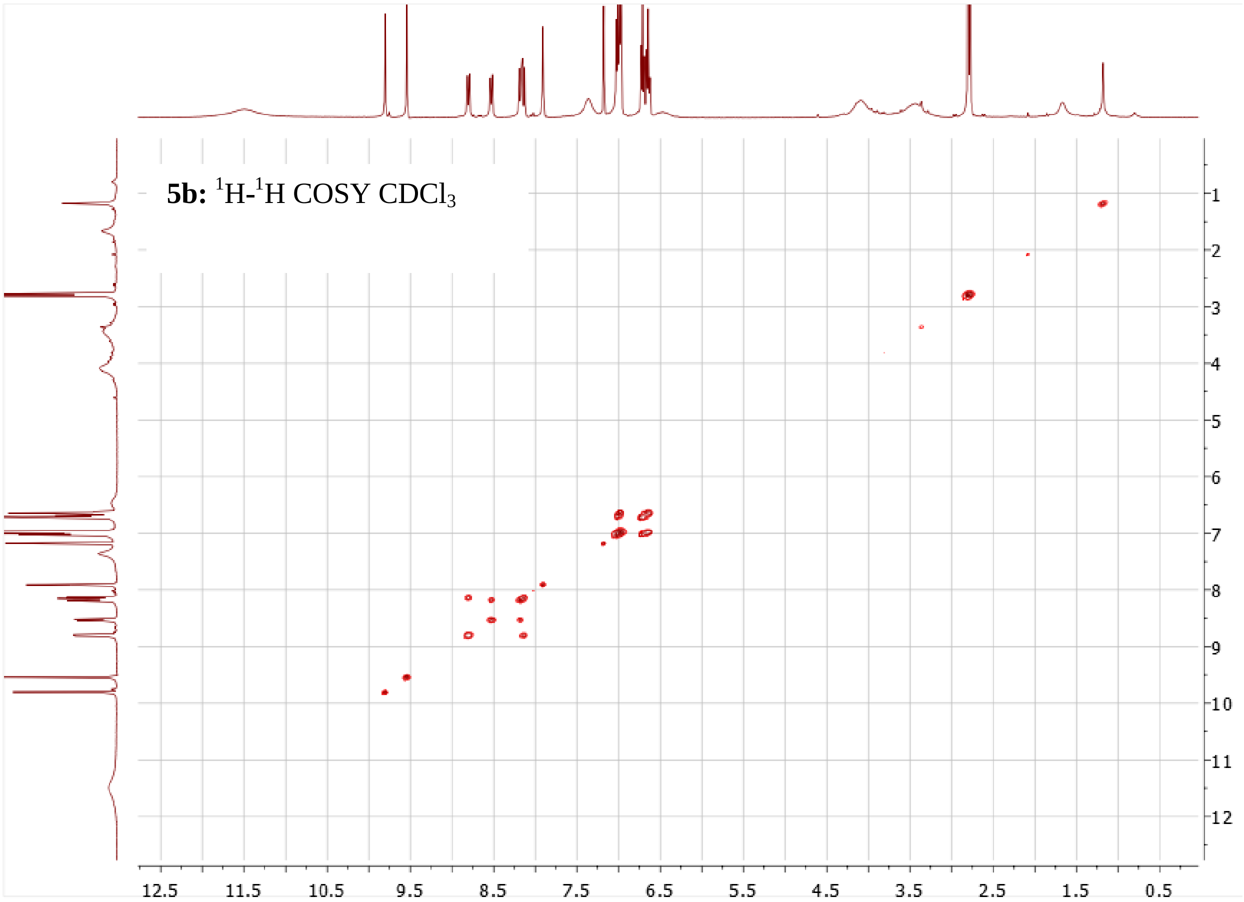

Spectrum S24. ^{1}H NMR (400 MHz, DMSO-d_6) of **5b**

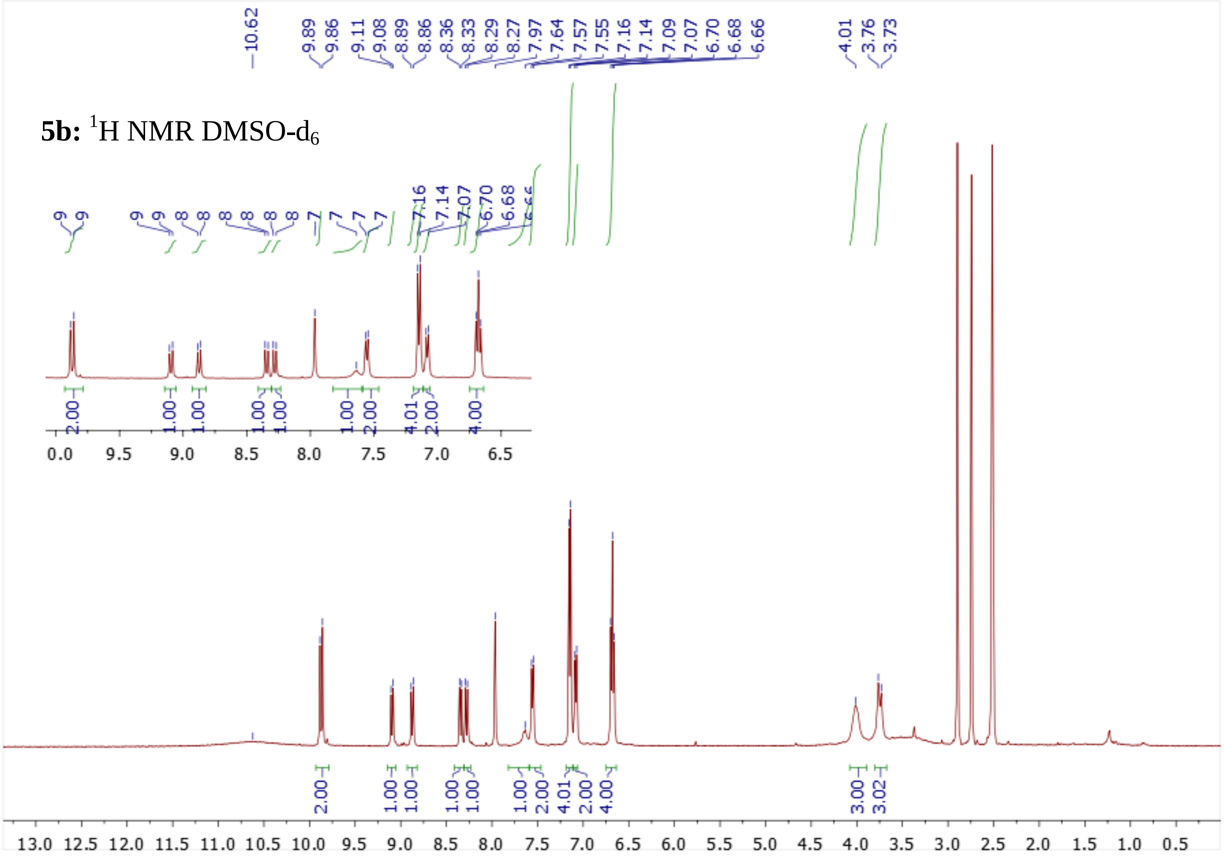

Spectrum S25. ^{1}H NMR (400 MHz, DMSO-d6) of **5b** with addition of $AlCl_3$ (10 equiv) in D_2O

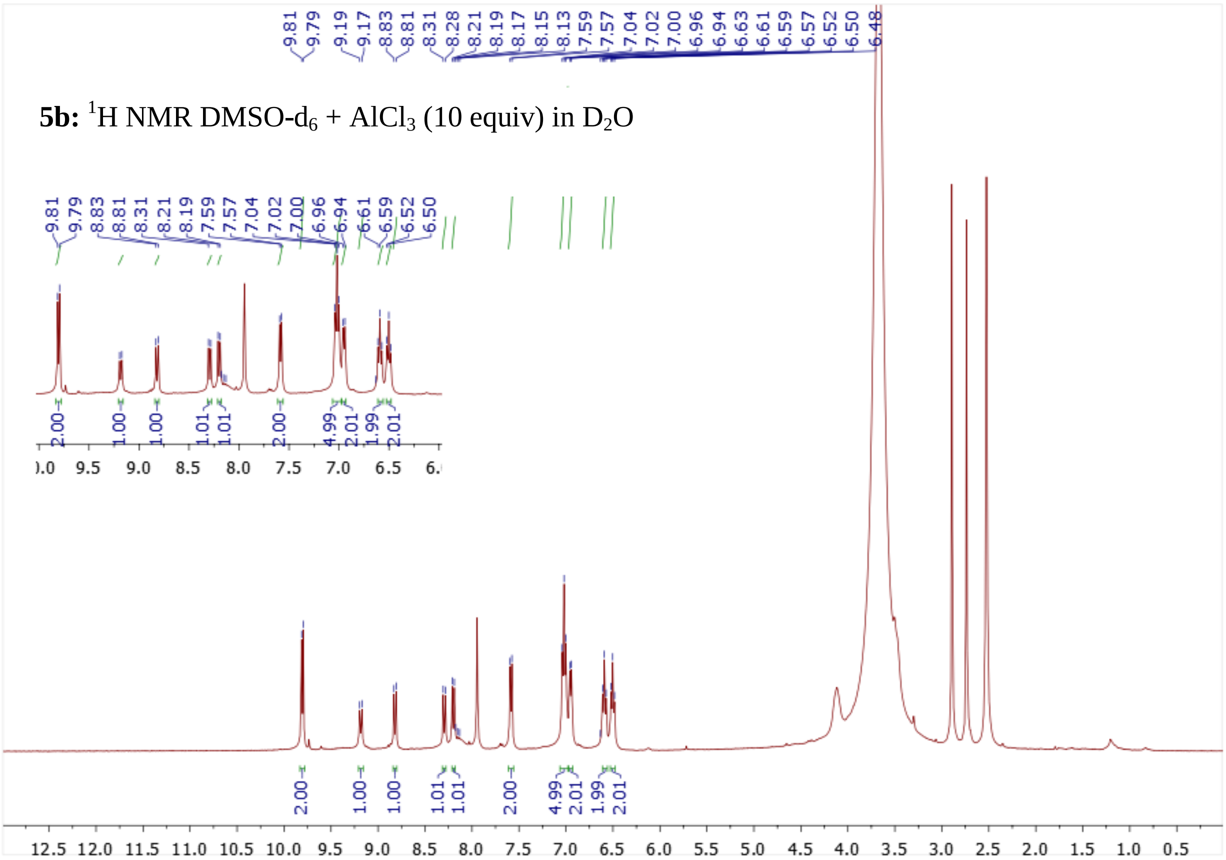

Spectrum S26. ^{1}H-^{1}H COSY NMR (DMSO-d6) of **5b** with addition of $AlCl_3$ (10 equiv) in D_2O

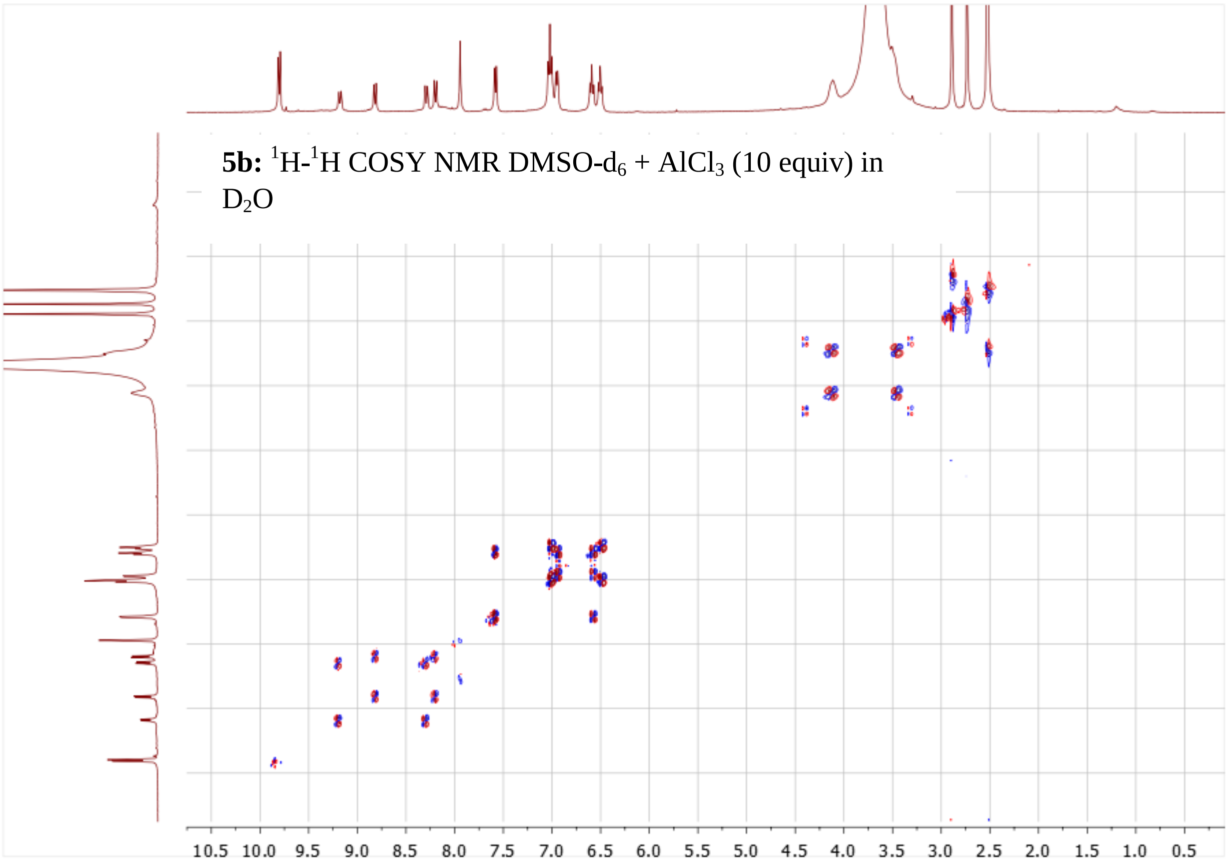

Spectrum S27. ^{1}H-^{1}H NOESY NMR (DMSO-d6) of **5b** with addition of $AlCl_3$ (10 equiv) in D_2O

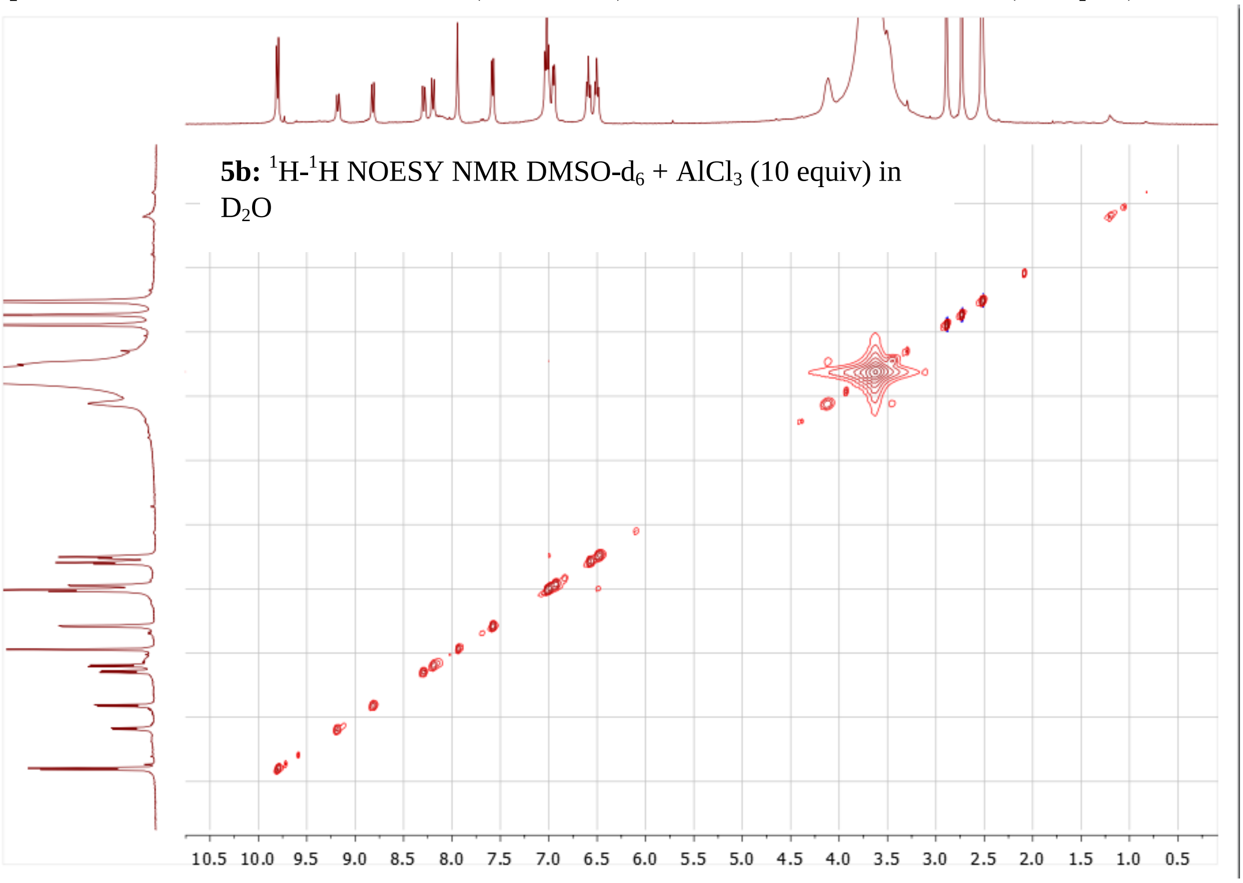

Spectrum S28. $^{13}C\{^{1}H\}$ NMR (100 MHz, DMSO-d6) of **5b** with addition of $AlCl_3$ (10 equiv) in D_2O

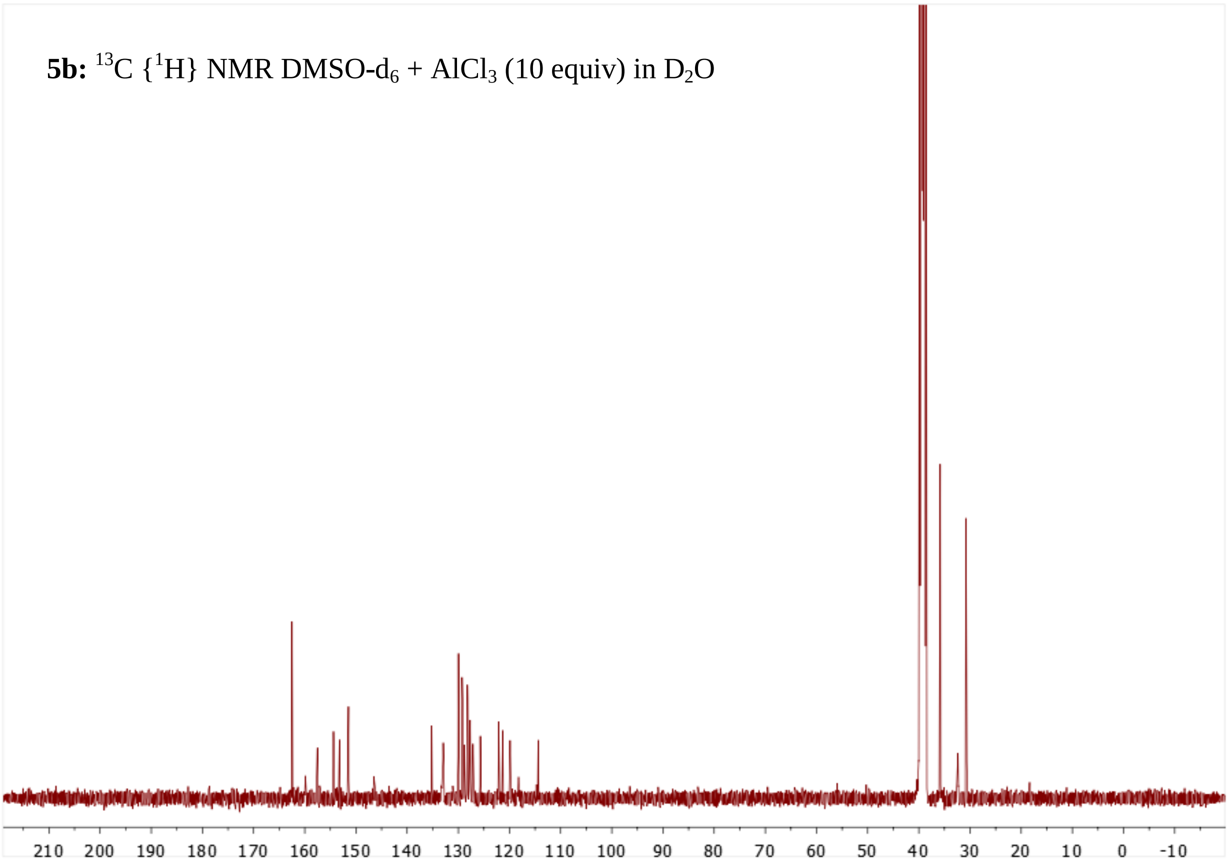

Spectrum S29. ^{1}H-^{13}C HSQC NMR (DMSO-d6) of **5b** with addition of $AlCl_3$ (10 equiv) in D_2O

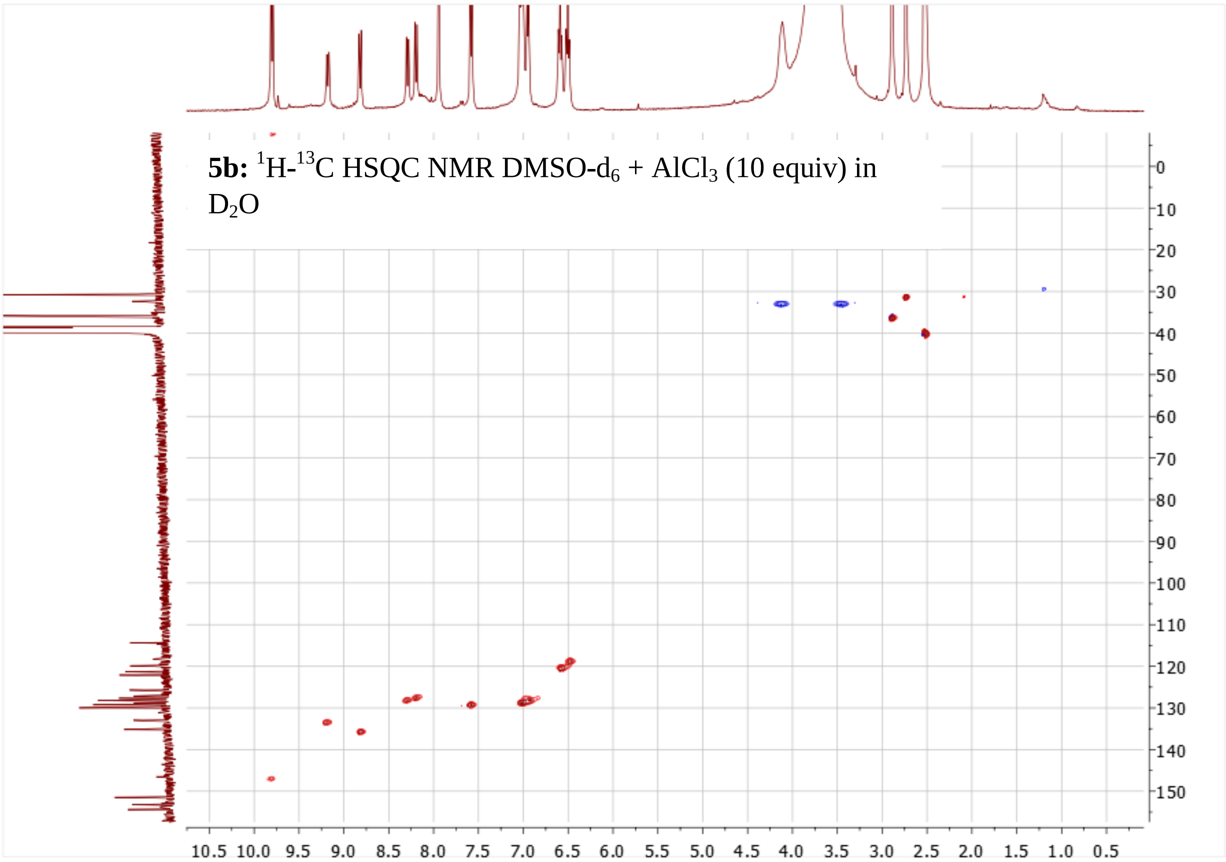

Spectrum S30. ^{1}H-^{13}C HMBC NMR (DMSO-d_6) of **5b** with addition of $AlCl_3$ (10 equiv) in D_2O

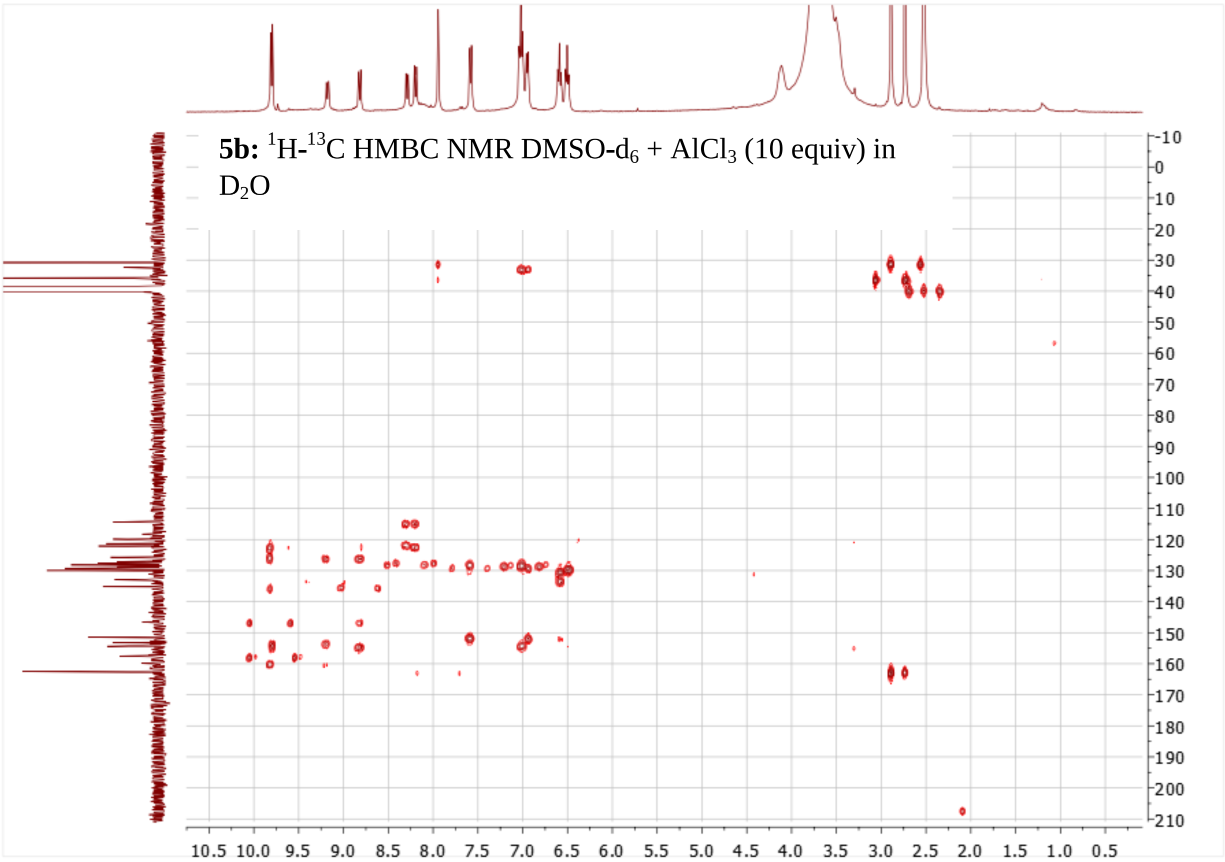

In order to clarify the reasons of changes in the NMR spectra (complex formation or conformational transformation), the control ^{1}H NMR experiment without Al^{3+} ions in the DMSO-d_6/D_2O mixture was carried out

Spectrum S31. ^{1}H NMR (DMSO-d_6/D_2O mixture) of **5b**

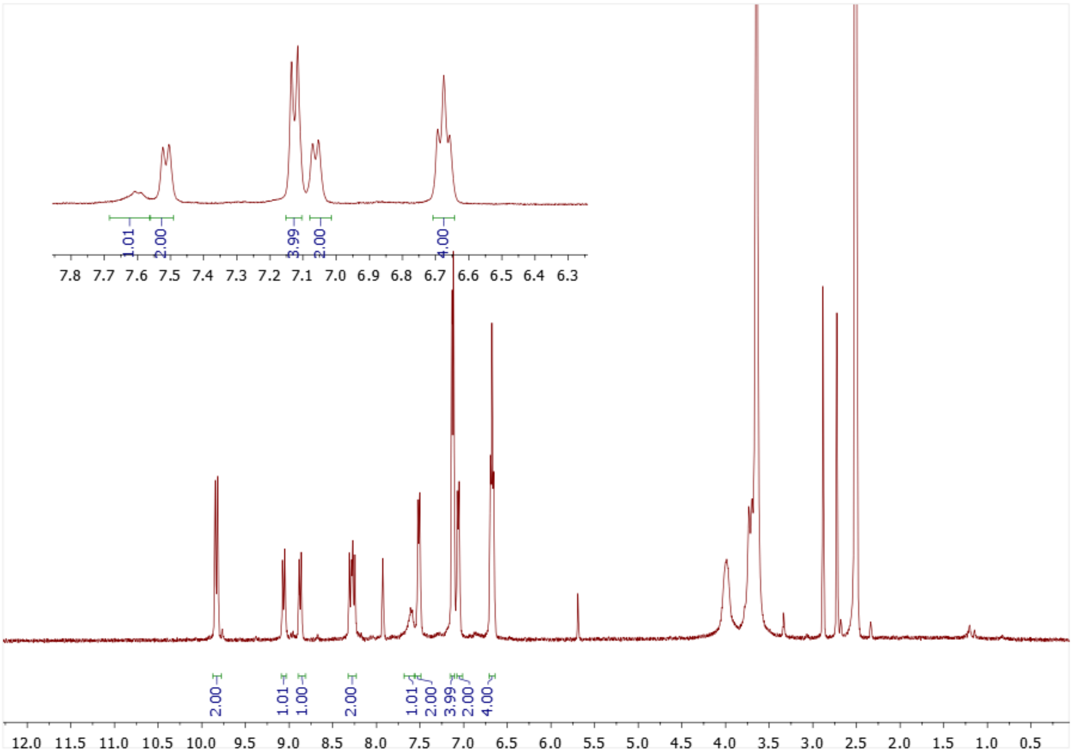

5. **Photophysical studies**

5.1. Absorbance spectra

Spectrum S32. Absorbance of **4a** (C = 10^{-5} M in THF)

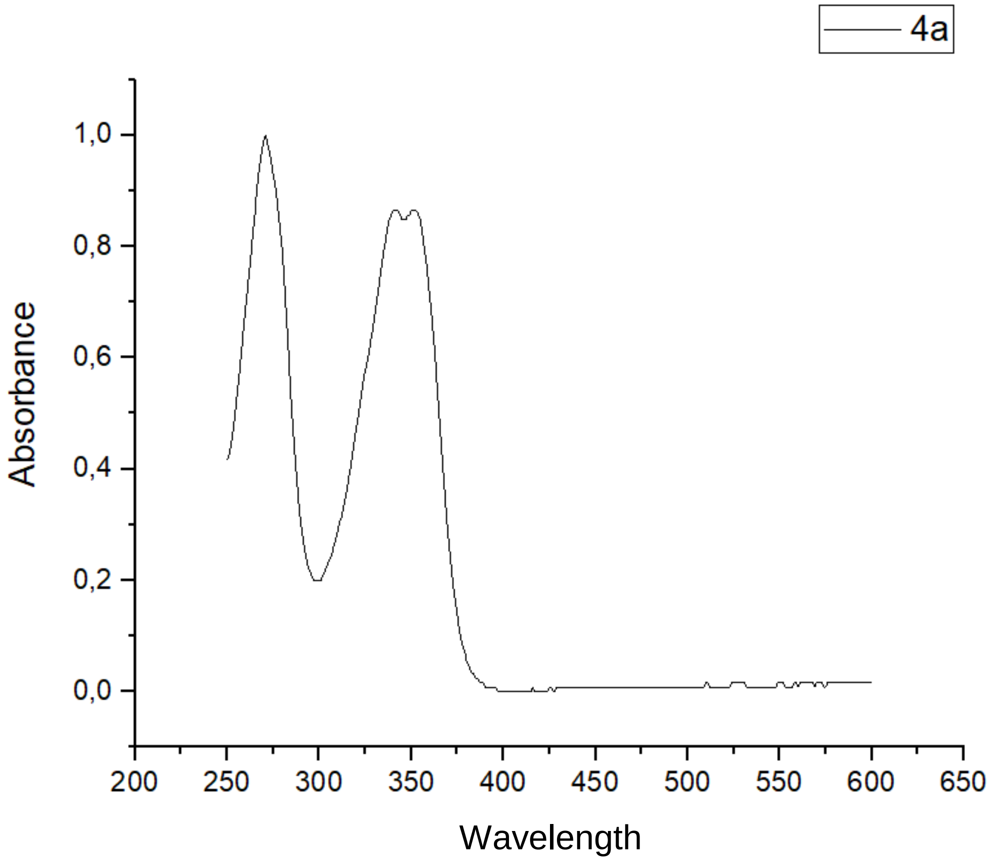

Spectrum S33. Absorbance of **4b** (C = 10^{-5} M in THF)

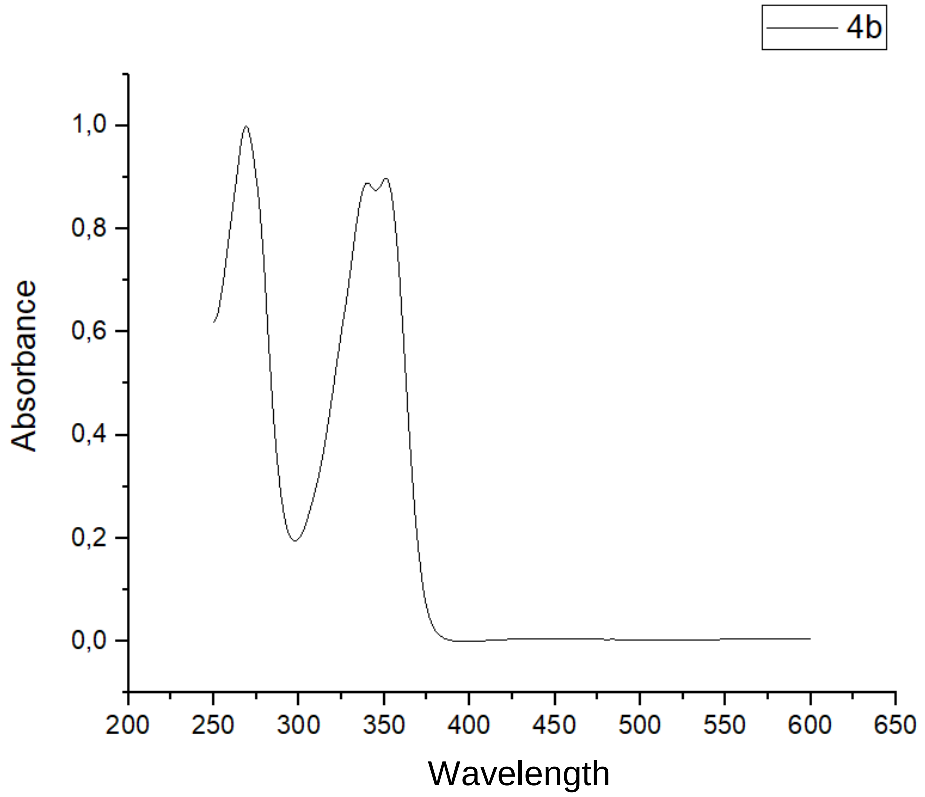

Spectrum S34. Absorbance of **5a** (C = 10^{-5} M in THF)

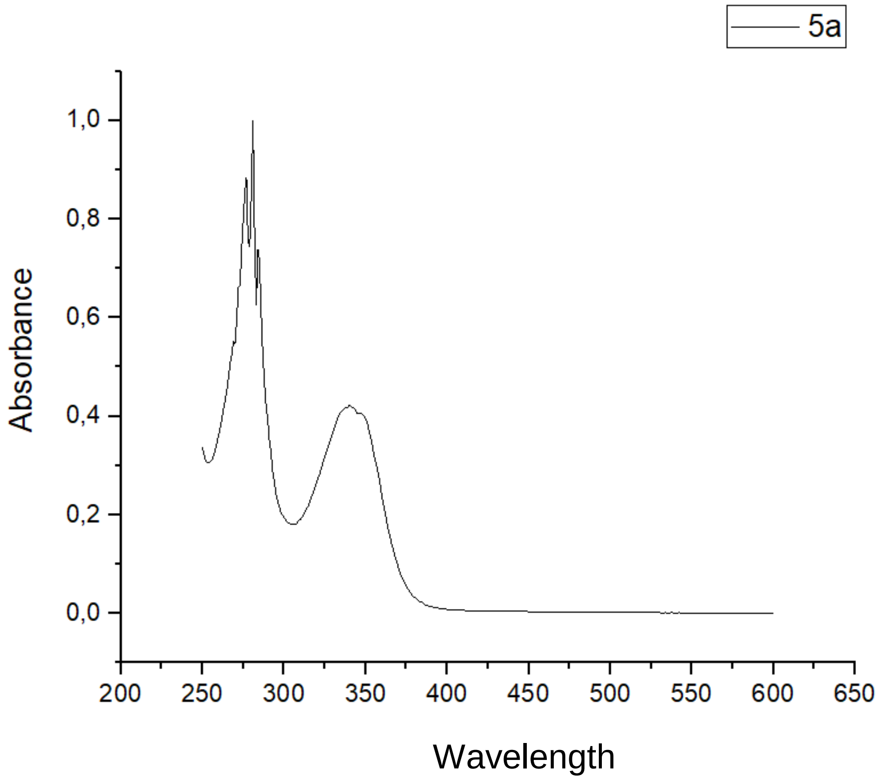

Spectrum S35. Absorbance of **5b** ($C = 10^{-5}$ M in THF)

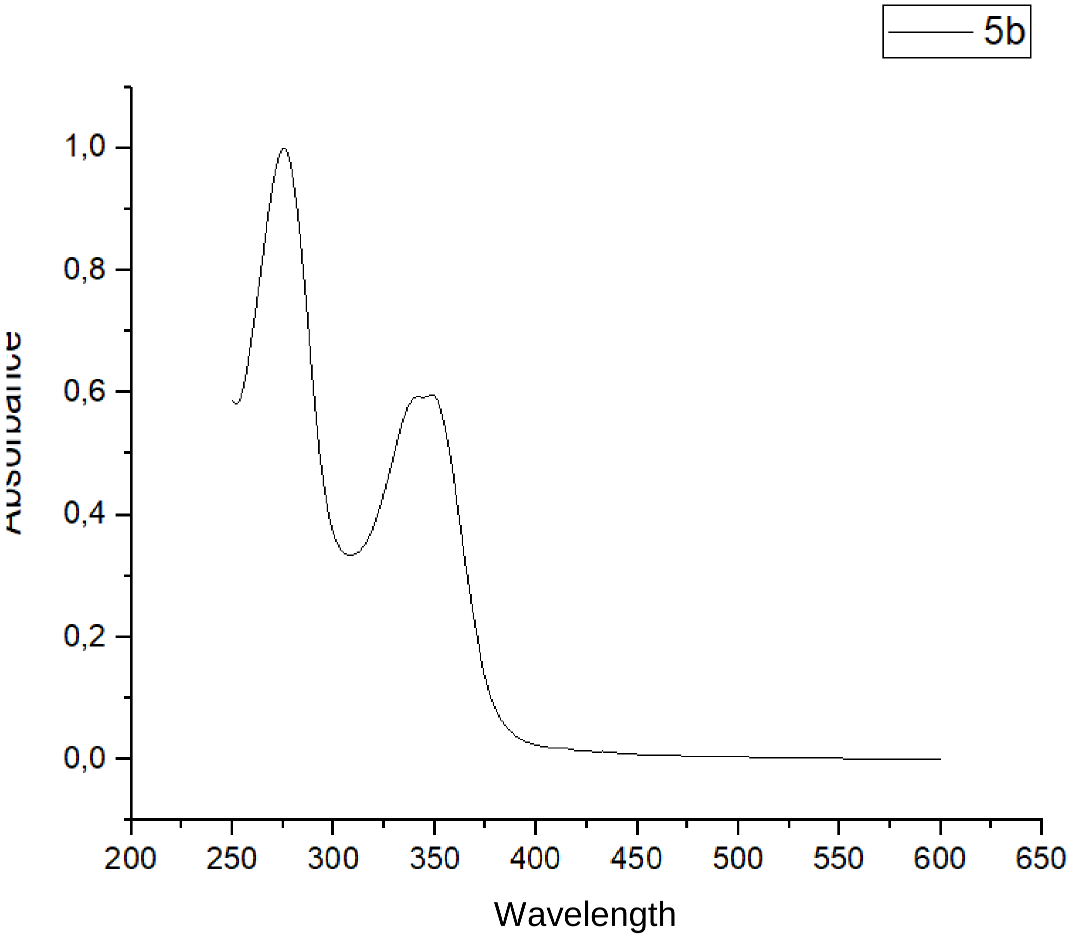

5.2. Emission spectra

Spectrum S36. Emission of **4a** (C = 10^{-5} M in THF) λ_{ex} = 352 nm

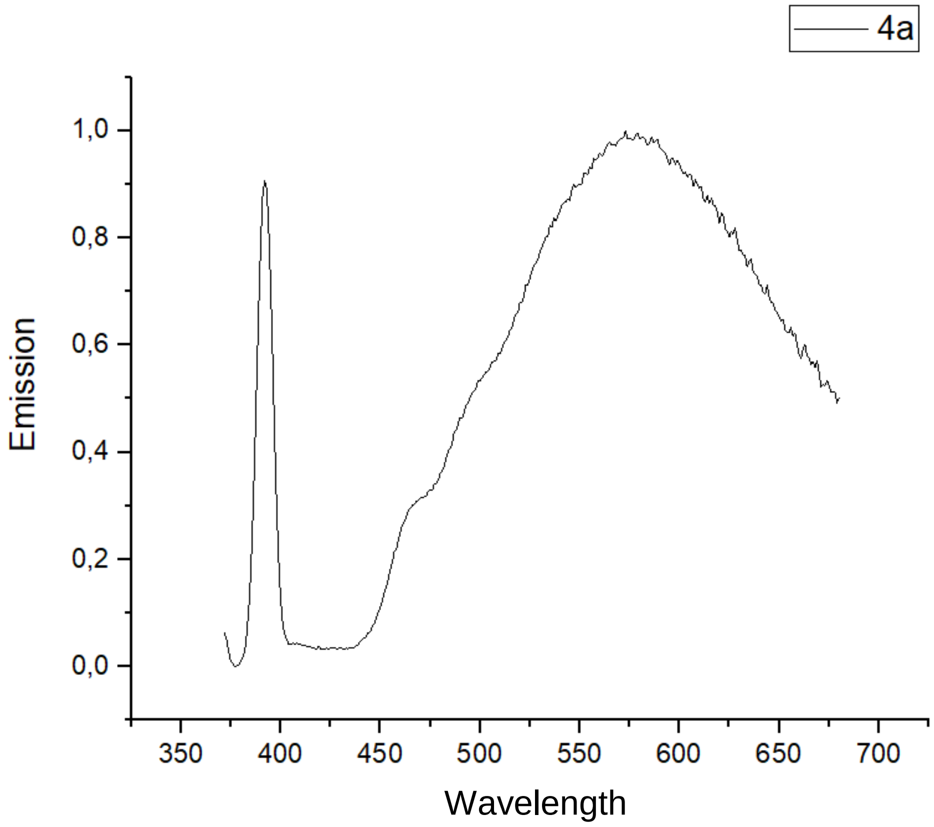

Spectrum S37. Emission of **4b** (C = 10^{-5} M in THF) λ_{ex} = 351 nm

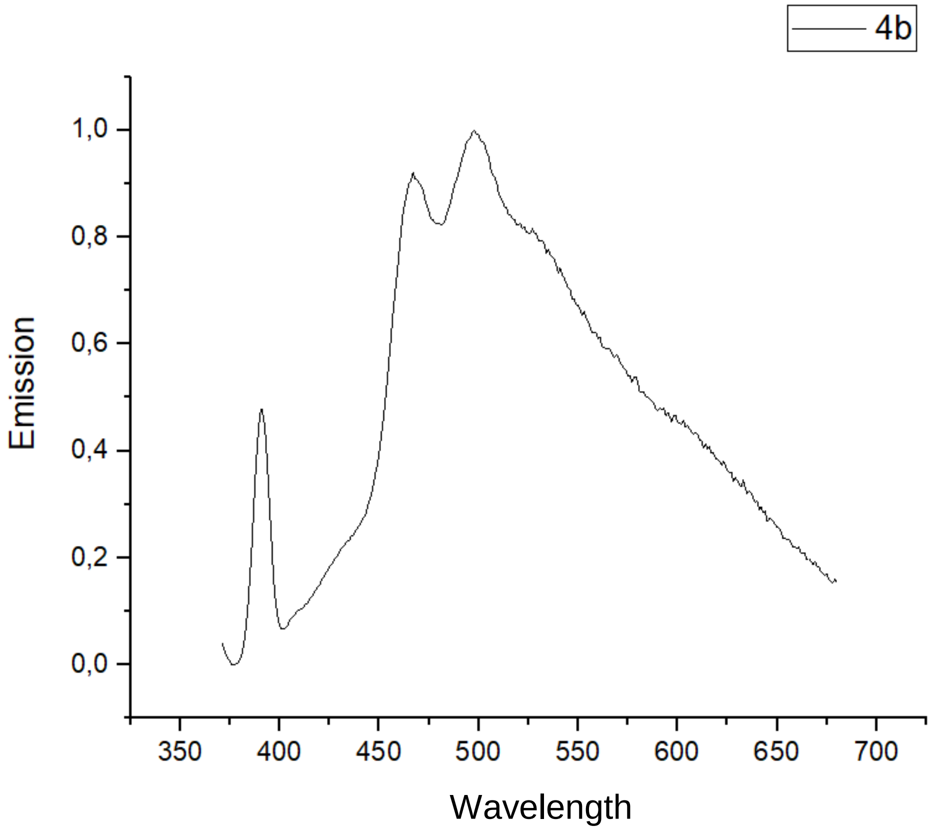

Spectrum S38. Emission of **5a** (C = 10^{-5} M in THF) λ_{ex} = 341 nm

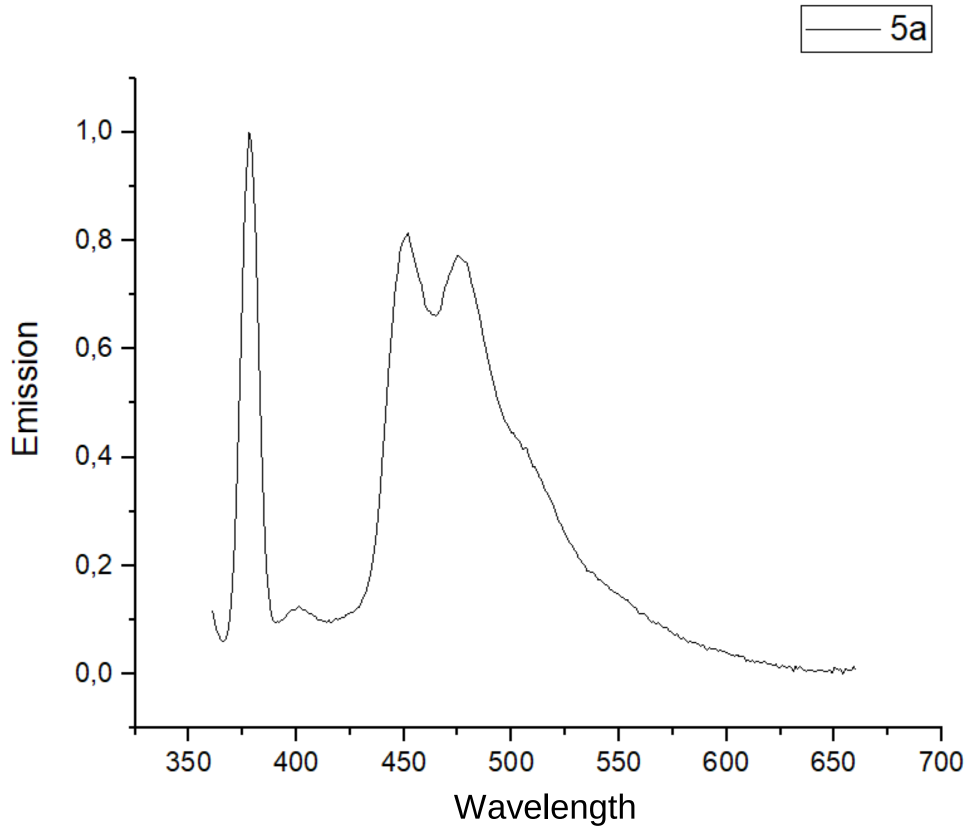

Spectrum S39. Emission of **5b** (C = 10^{-5} M in THF) λ_{ex} = 350 nm

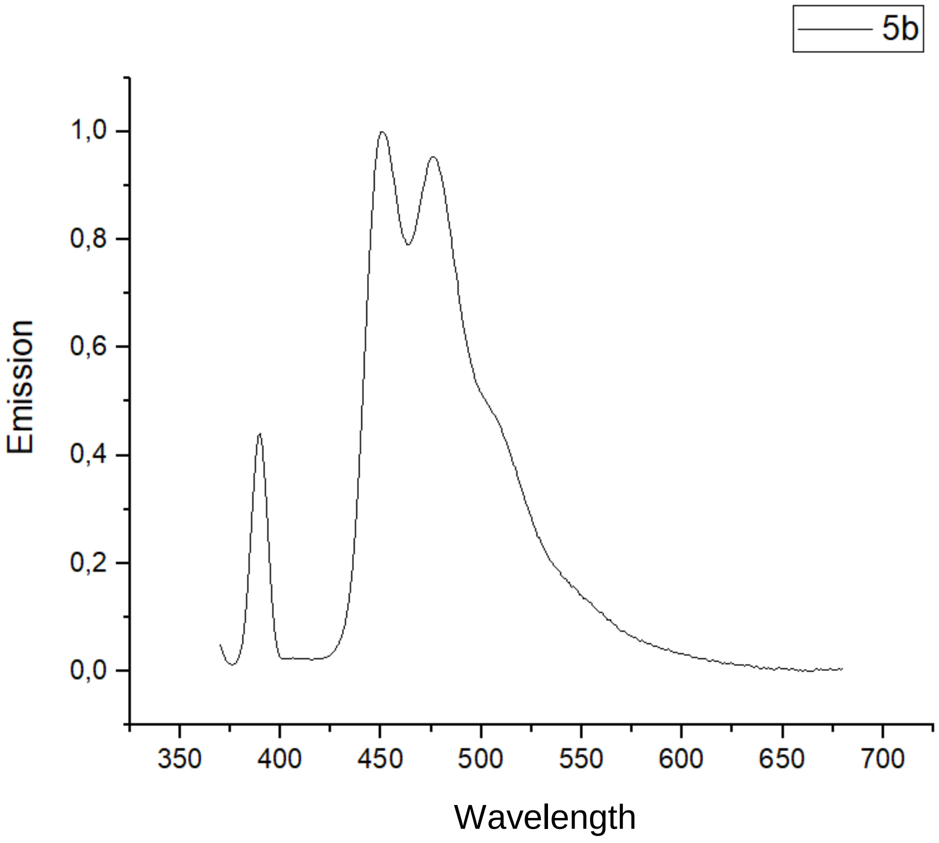

5.3. Emission spectra in the presence of various metal ions

Spectrum S40. Emission of **4a** (C = 10^{-5} M in THF) with the presence of various metal ions (10 equiv in H_2O) λ_{ex} = 352 nm

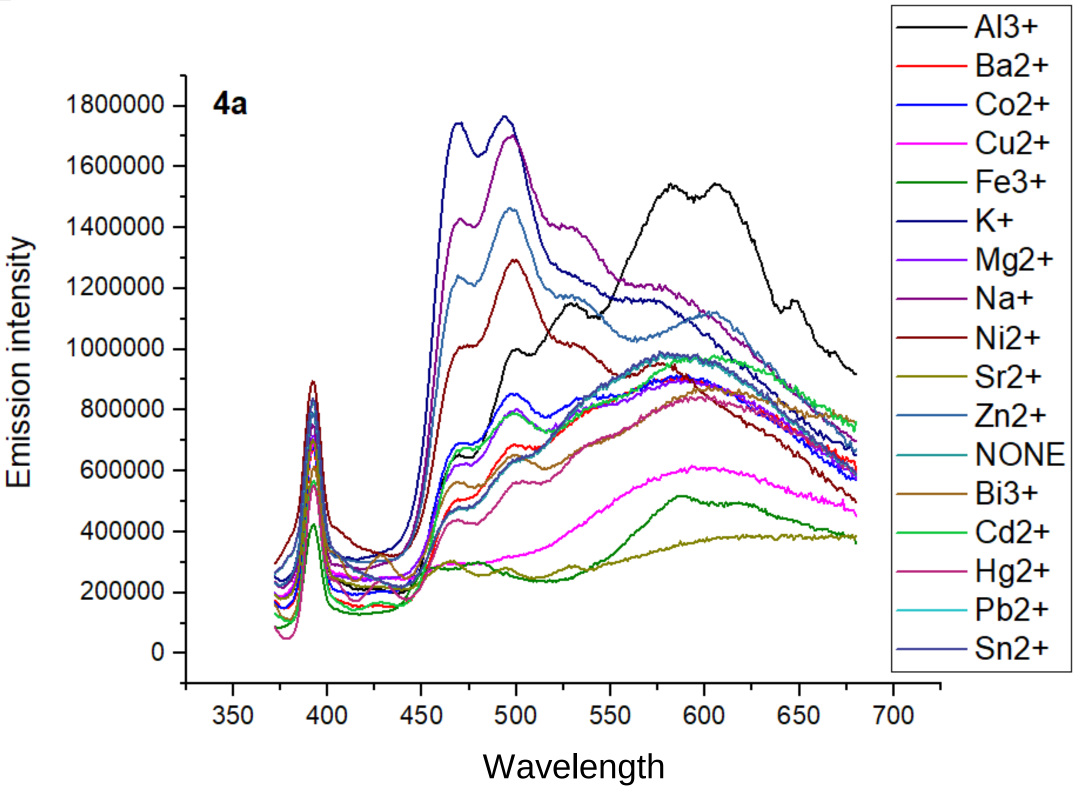

Spectrum S41. Emission of **4b** (C = 10^{-5} M in THF) with the presence of various metal ions (10 equiv in H_2O) λ_{ex} = 351 nm

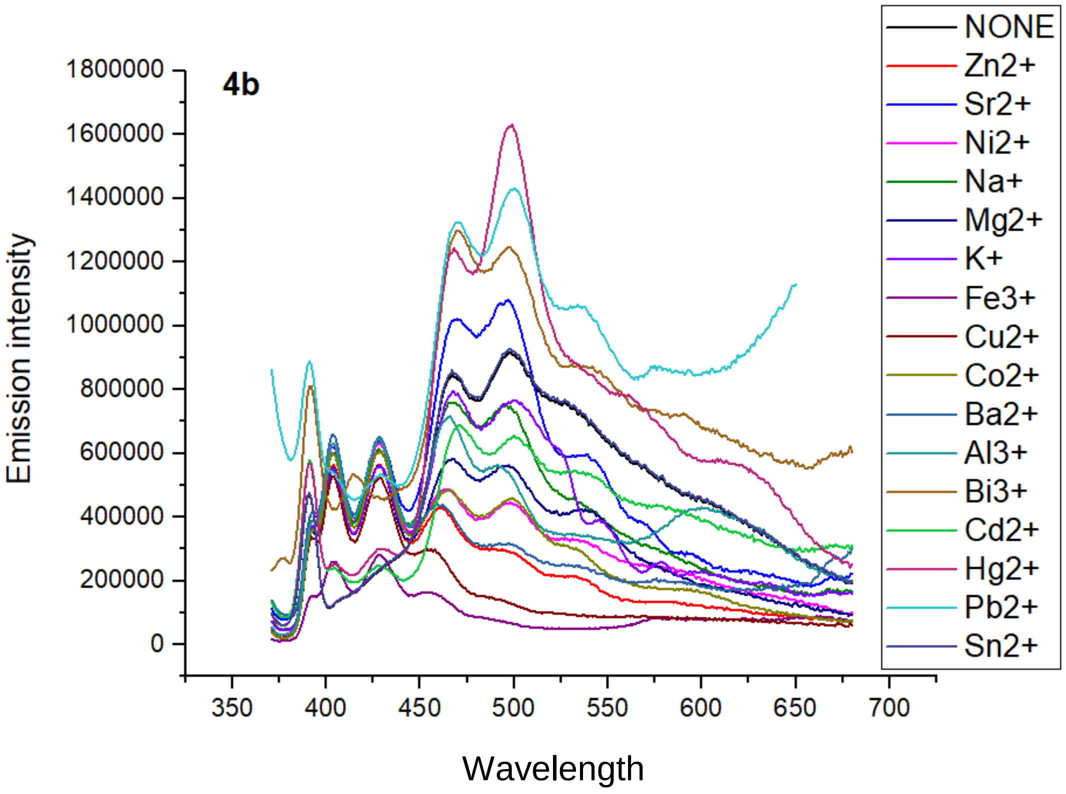

Spectrum S42. Emission of **5a** (C = 10^{-5} M in THF) with the presence of various metal ions (10 equiv in H_2O) λ_{ex} = 341 nm

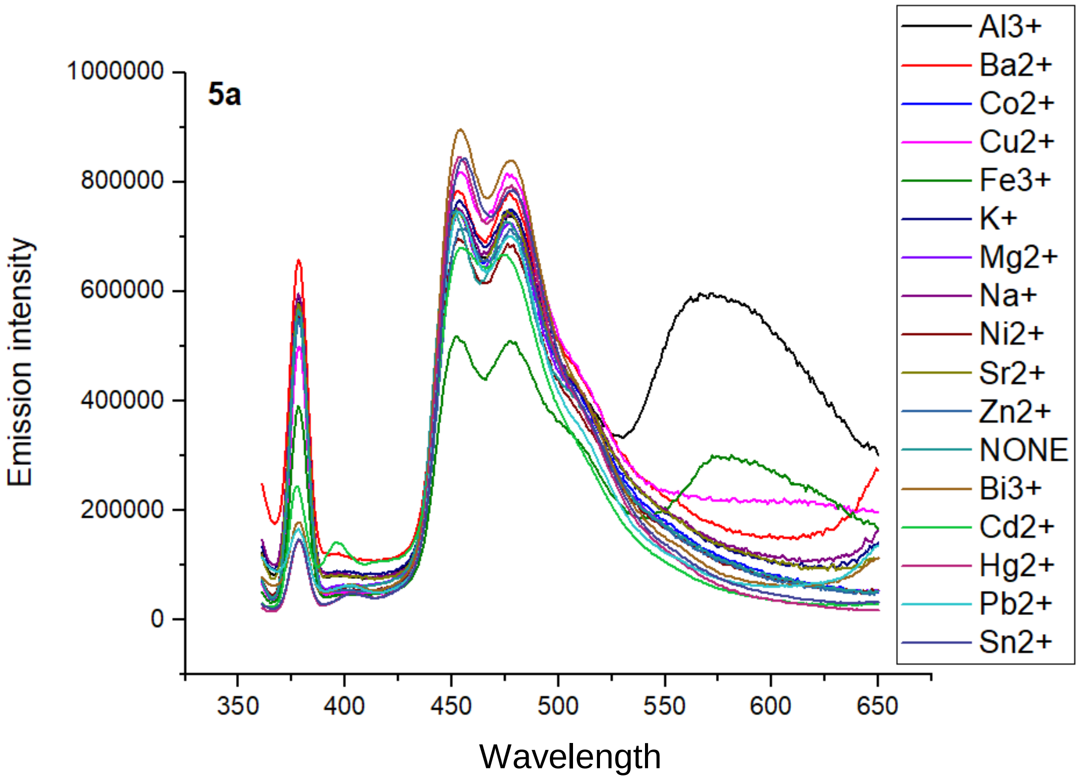

Spectrum S43. Emission of **5b** (C = 10^{-5} M in THF) with the presence of various metal ions (10 equiv in H_2O) λ_{ex} = 350 nm

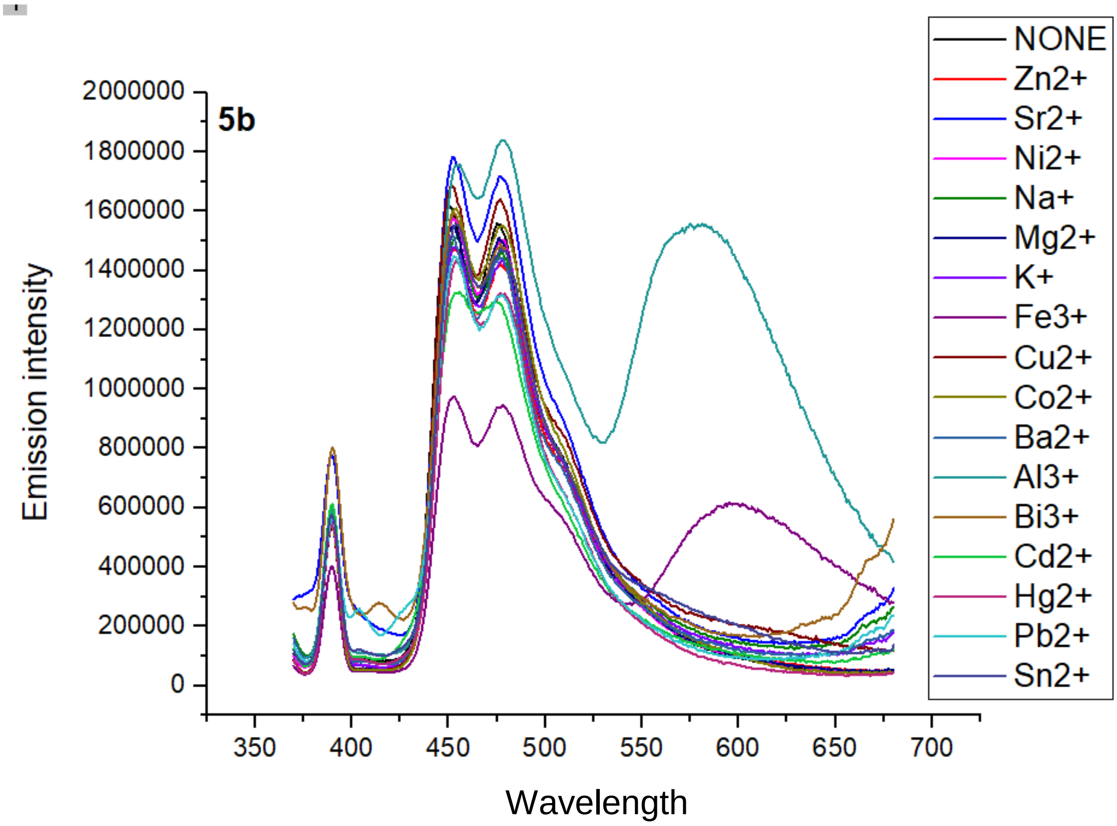

6. Determining the stoichiometry of ligand: metal complex

The Job's method[1] was applied to determine the stoichiometry of **5b**:Al^{3+} and **5b**:Fe^{3+} complexes.

Figure S1. Job's plot for stoichiometry determination of **5b**:Al^{3+}complex, λ_{ex} = 350 nm, λ_{em} = 570 nm.

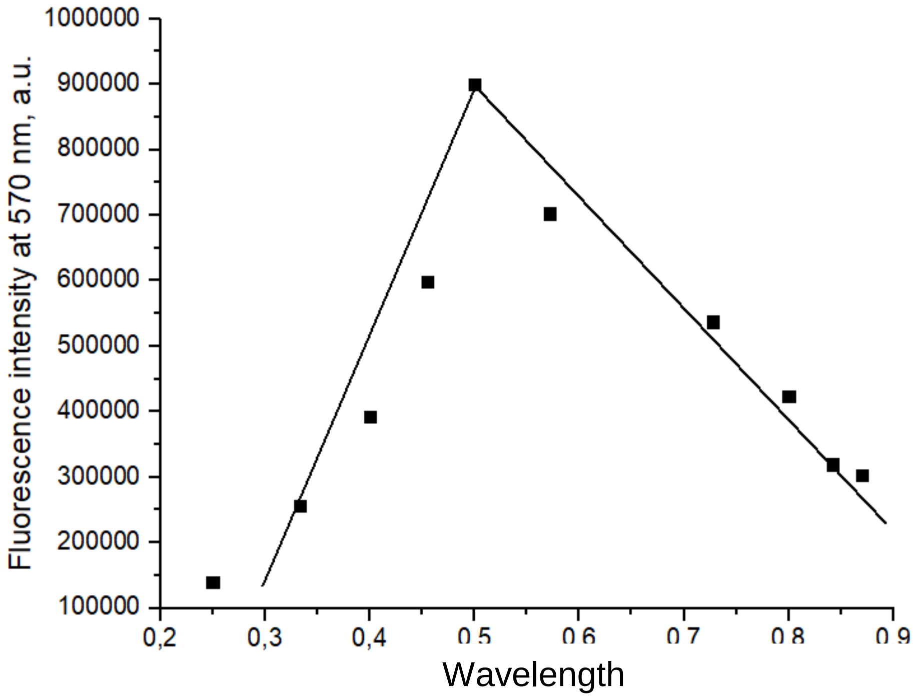

Figure S2. Job's plot for determination stoichiometry of **5b**:Fe^{3+} complex, λ_{ex} = 350 nm, λ_{em} = 570 nm.

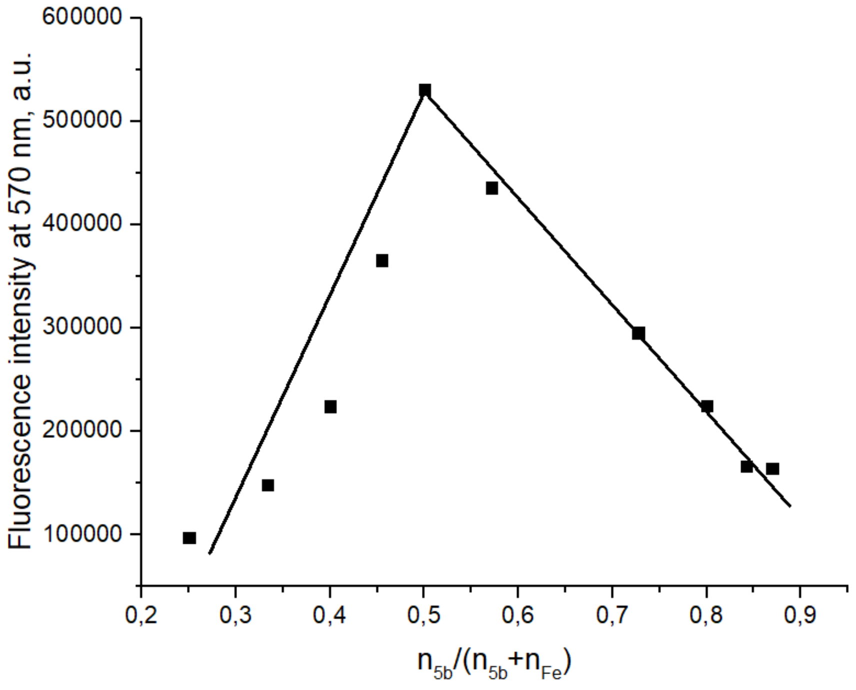

7. Binding constants measurement

Figure S3. Plot of emission intensity of **5b** with increasing concertation of Al^{3+} in THF, λ_{ex} = 350 nm, λ_{em} = 570 nm

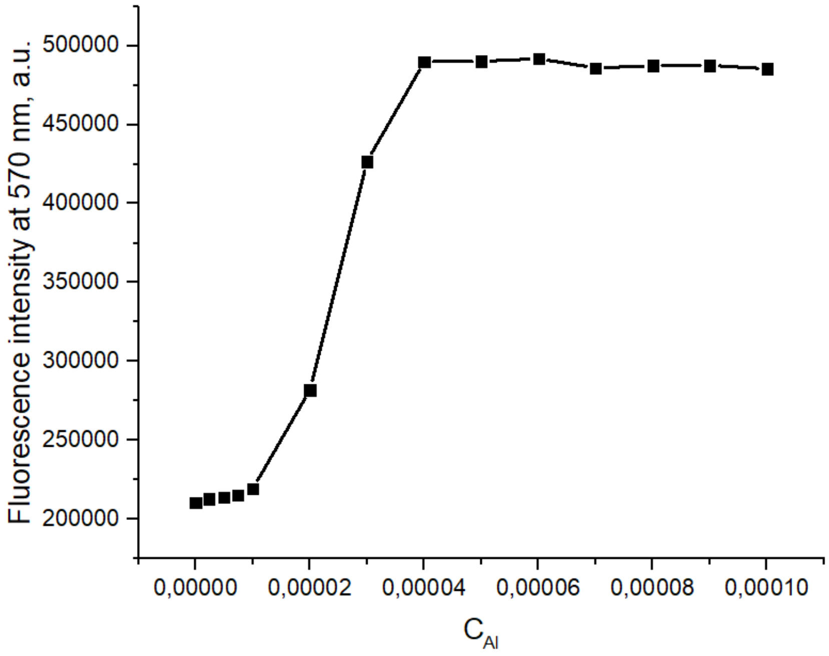

Figure S4. Plot of emission intensity of **5b** with increasing concertation of Fe^{3+} in THF, λ_{ex} = 350 nm, λ_{em} = 570 nm

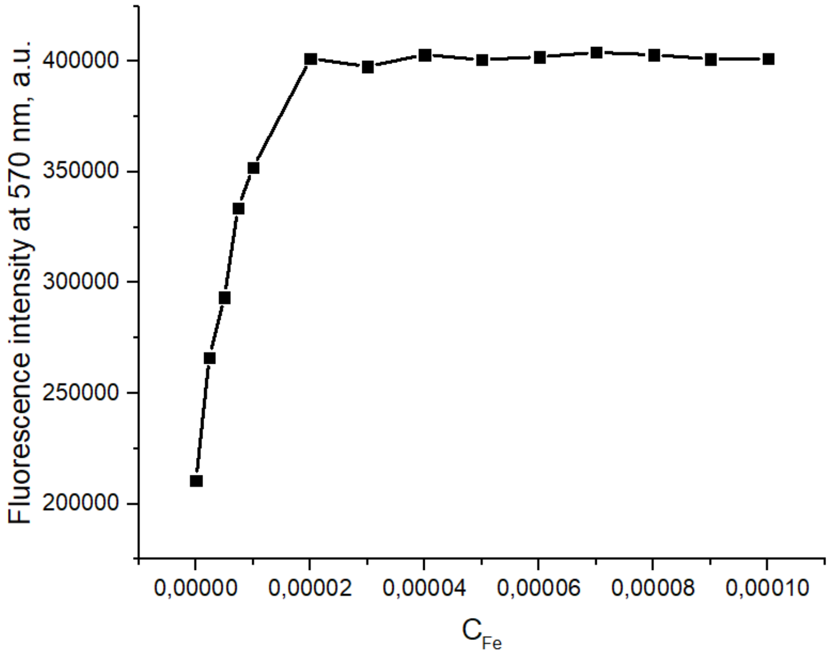

Binding interactions of **5b** with Al^{3+} and Fe^{3+} cations in THF with addition of various concertation of guests in H_2O have been estimated using the modified Benesi–Hildebrand equation.[2]

$(F_{max} - F_0) / (F_x - F_0) = 1 + (1/K) (1/[M]^n)$, where F_0, F_x and F_{max} are the emission intensities of **5b** in the absence of Al^{3+} or Fe^{3+}, at an intermediate Al^{3+} and Fe^{3+}, and at a concentration of complete interaction, respectively, where K is the binding constant, M is the concentration of Al^{3+} or Fe^{3+} and n is the number of Al^{3+} or Fe^{3+} ions bound per **5b** (here, n = 1).

Emission intensities of **5b** (10 μM) in presence of different Al^{3+} or Fe^{3+} concentrations, namely 2.33, 5, 7.33, 10, 30, 50, 70, 90 μM have been used for calculation of binding constant.

The equation (example for Al^{3+}) used is:

$(F_{max} - F_0) / (F_x - F_0) = 1+ (1/K[M]^n)$, where F_{max} = 492880 a.u., F_0 = 210512 a.u., n = 1.

When 30 μM Al^{3+} is added to **5b** (10 μM), Fx = 426766 a.u.,

Now, $(F_{max}-F_0) / (F_x-F_0)$ = (492880 - 210512) / (426766- 210512) and 1/ [M] = 1/(30)

or, 1.3057 = 1 + 1/ 30K

Similar calculations were performed to other concentrations.

Thus, plot of $(F_{max} - F_0) / (F_x - F_0)$ *vs.* 1/ [M] based on Y = 1+ P × X, yields the slope, P = 439.97 for Al^{3+} and 6.13 for Fe^{3+}.

So, $K(Al^{3+}) = 2.27 \cdot 10^3\ M^{-1}$; $K(Fe^{3+}) = 1.63 \cdot 10^5\ M^{-1}$.

Figure S5. Benesi–Hildebrand equation for determining the binding constant of **5b** with Al^{3+} using the fluorescent titration, λ_{ex} = 350 nm, λ_{em} = 570 nm

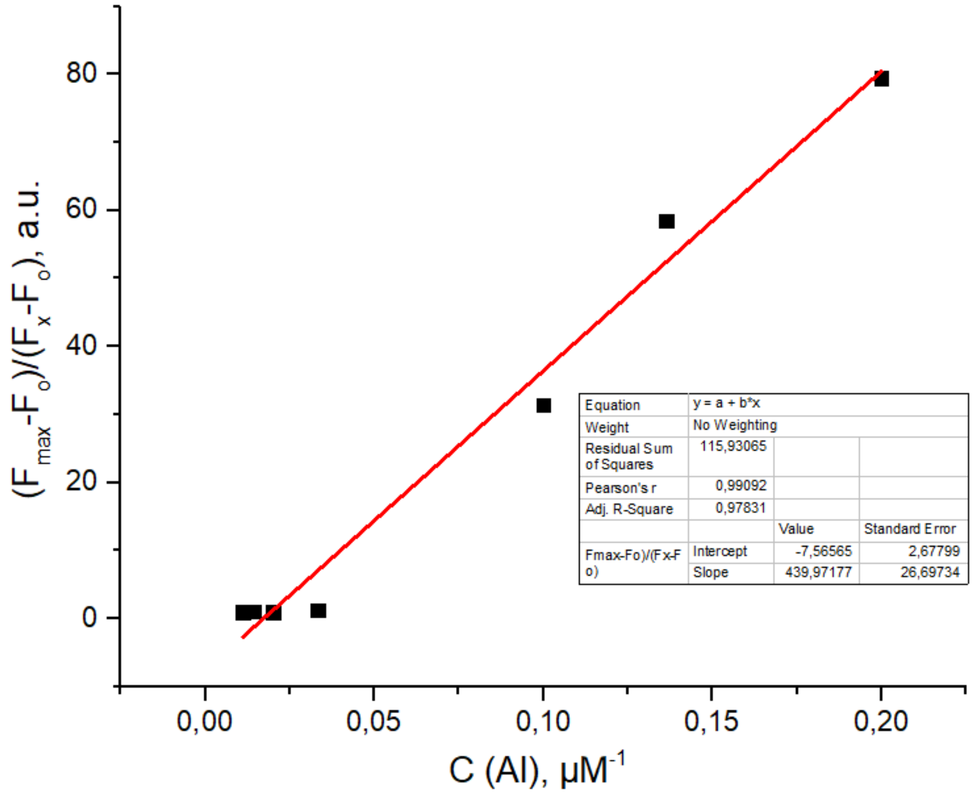

Figure S6. Benesi–Hildebrand equation for determining binding constant of **5b** with Fe^{3+} using the fluorescent titration, λ_{ex} = 350 nm, λ_{em} = 570 nm

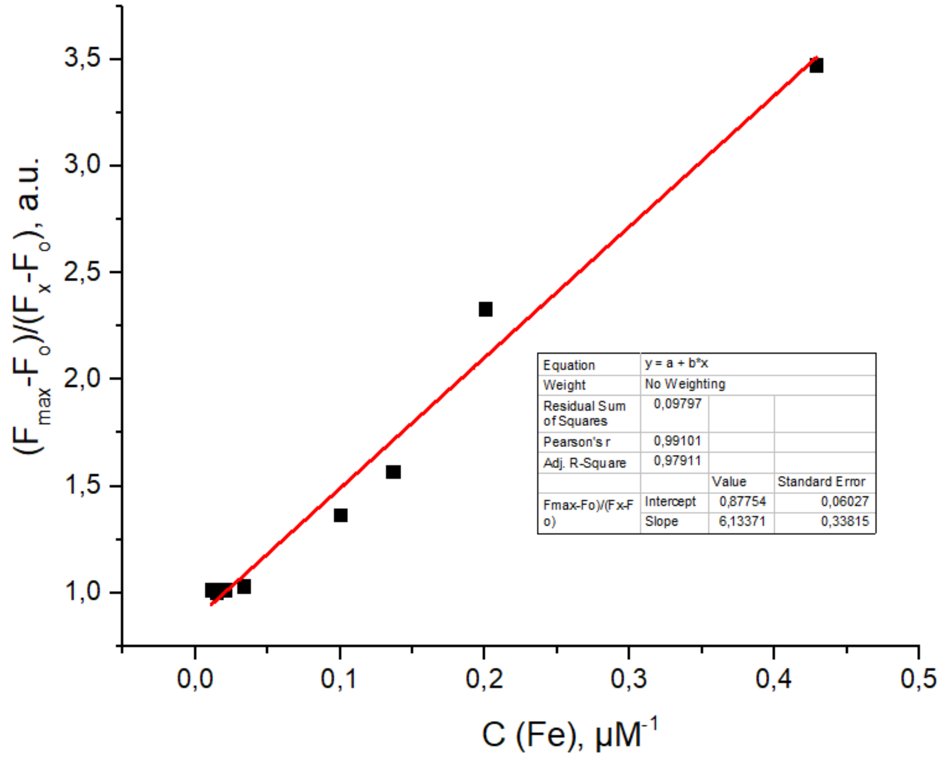

8. Emission spectra depending on pH

Spectrum S44. Emission intensity of **5b** in different pH, λ_{ex} = 350 nm, λ_{em} = 570 nm

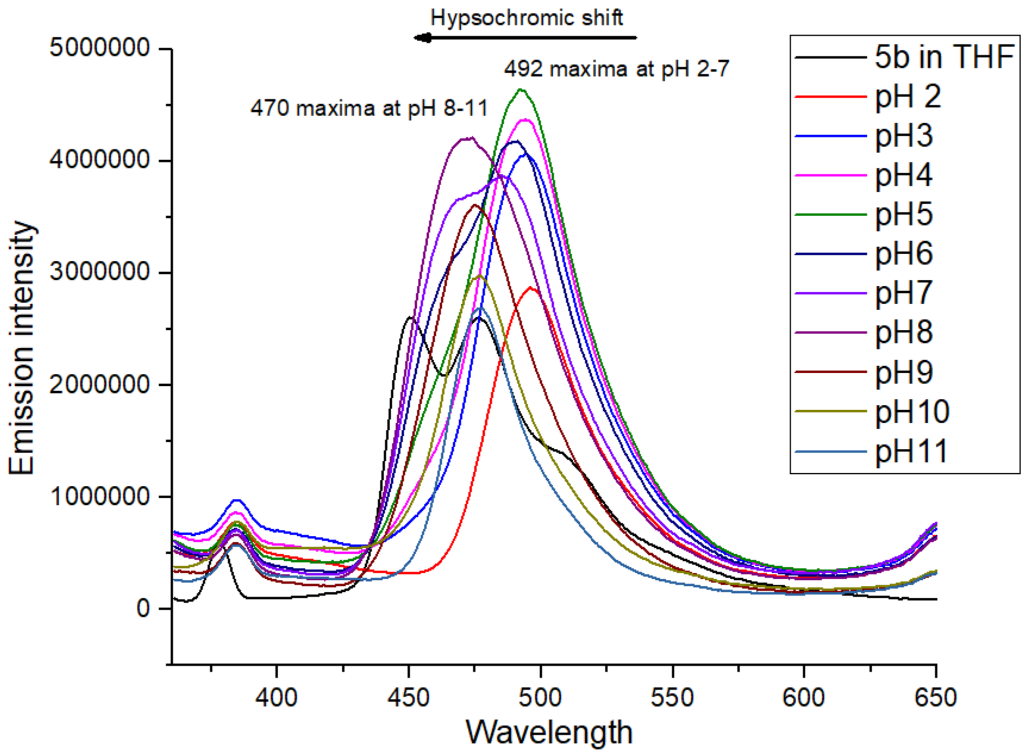

Spectrum S45. Emission intensity of **5b** in different pH with addition of 10 equiv of Al^{3+}, λ_{ex} = 350 nm, λ_{em} = 570 nm

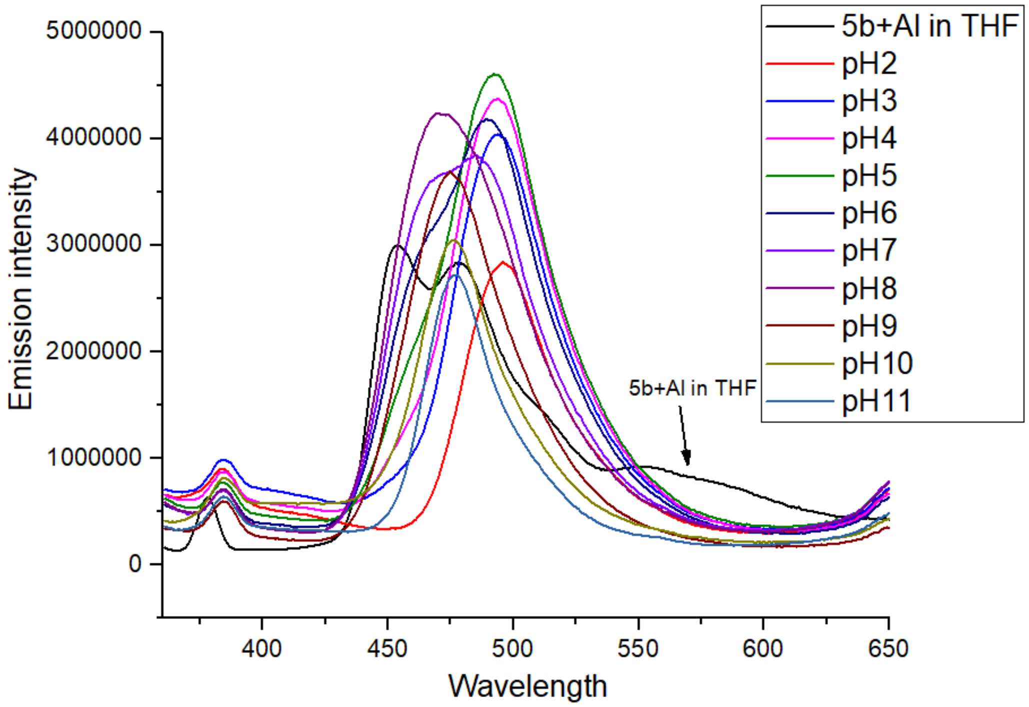

Spectrum S46. Emission intensity of **5b** in different pH with addition of 10 equiv of Al^{3+} and Fe^{3+}, λ_{ex} = 350 nm, λ_{em} = 570 nm

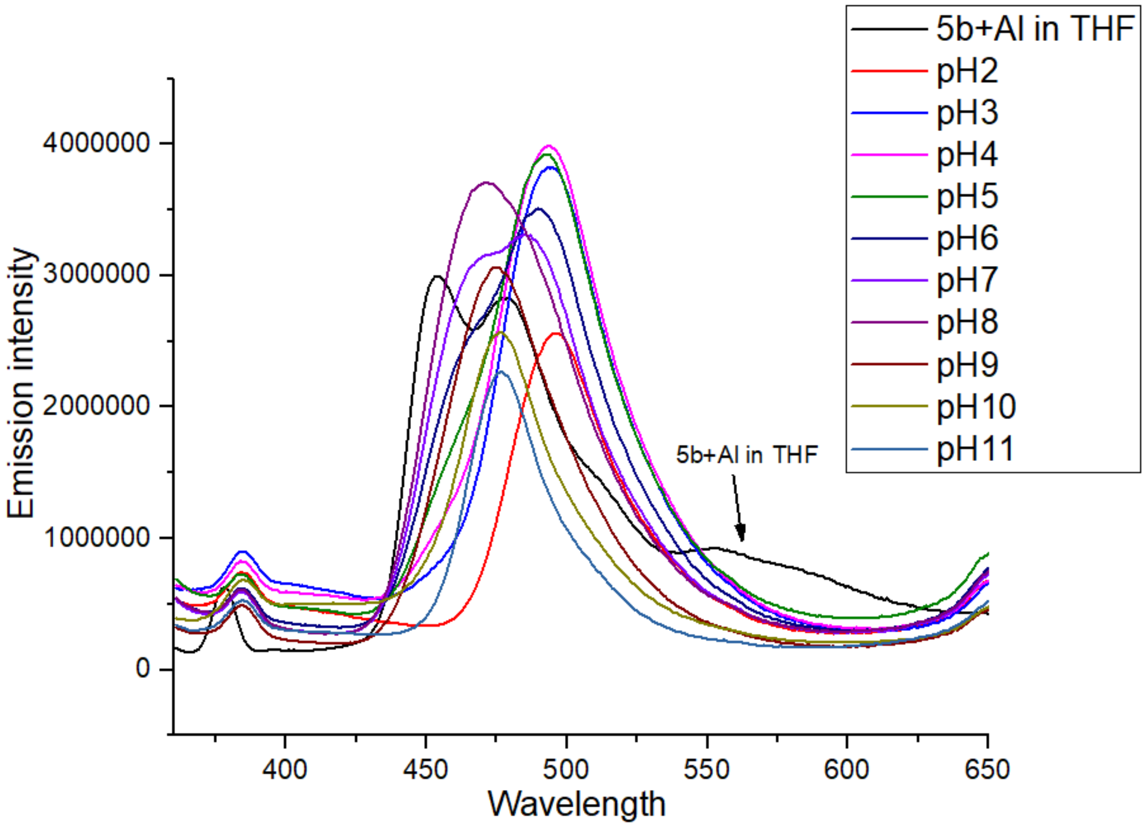

9. References

1. J. S. Renny, L.L. Tomasevich, E.H. Tallmadge, D.B. Collum, *Angew. Chemie Int. Ed.*, 2013, **52 (46)**, 11998–12013.
2. (a) R.L. Scott, *Recl. des Trav. Chim. des Pays-Bas*, 1956, **75 (7)**, 787–789. (b) A. Sahana, A. Banerjee, S. Lohar, S. Panja, S. Kanti Mukhopadhyay, J. Sanmartín Matalobos, D. Das, *Chem. Commun.*, 2013, **49 (65)**, 7231-7233.
